# Supplementary material for: Conformation of the Human Immunoglobulin G2 Hinge Imparts Superagonistic Properties to Immunostimulatory Anticancer Antibodies
Source: Cancer Cell. 2015 Jan 12;27(1):138–48. doi: 10.1016/j.ccell.2014.11.001 (PMC4297290; doi:10.1016/j.ccell.2014.11.001)
Supplement: Document S1. Supplemental Experimental Procedures and Figures S1–S5 [file mmc1.pdf]

Cancer Cell, Volume *27*

Supplemental Information

Conformation of the Human Immunoglobulin G2 Hinge Imparts Superagonistic Properties to Immunostimulatory Anticancer Antibodies

Ann L. White, H.T. Claude Chan, Ruth R. French, Jane Willoughby, C. Ian Mockridge, Ali Roghanian, Christine A. Penfold, Steven G. Booth, Ali Dodhy, Marta E. Polak, Elizabeth A. Potter, Michael R. Ardern-Jones, J. Sjef Verbeek, Peter W.M. Johnson, Aymen Al-Shamkhani, Mark S. Cragg, Stephen A. Beers, and Martin J. Glennie

SUPPLEMENTAL DATA

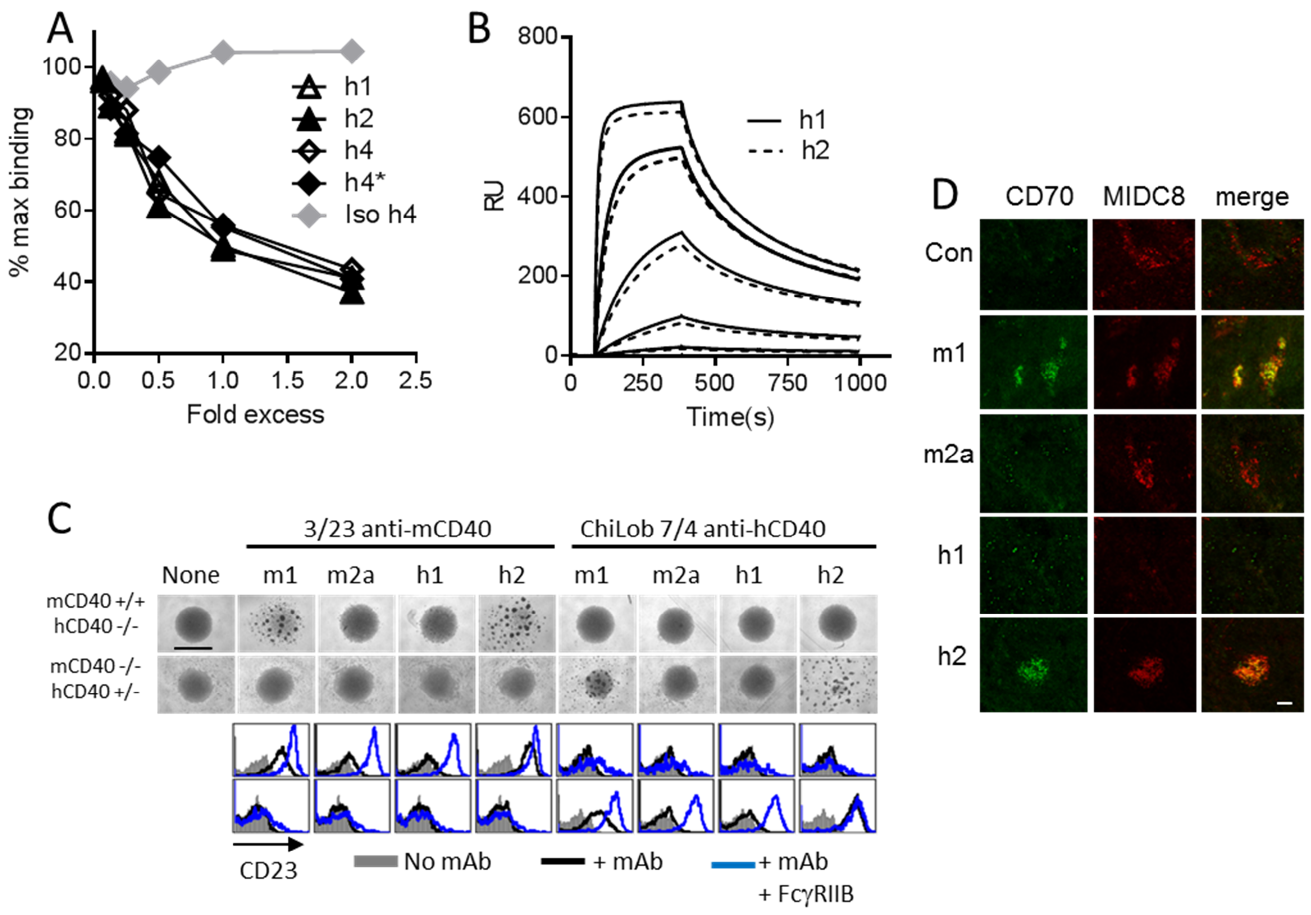

Figure S1. Related to Figure 1. Control experiments for effect of isotype on anti-CD40 activity.

(A) Purified hCD40Tg mouse B cells were incubated with 10 μg/ml ChiLob 7/4 h1-FITC pre-mixed with different concentrations of competing unlabelled ChiLob 7/4 mAb of different human isotypes. Flow cytometry was used to determine the level of ChiLob 7/4-FITC binding and is expressed as the % maximum MFI (no competing mAb present). A non-targeted human IgG4 isotype mAb (grey diamonds) was included as a non-competing control. **(B)** hCD40 was immobilised at 1000 RU and ChiLob 7/4 h1 (solid line) or h2 (broken line) Fab' fragments flowed over the chip at 640, 128, 25.6 and 1.024 nM. Affinities were calculated by fitting a 1:1 binding model and were 10.0 and 10.2 nM for h1 and h2, respectively. **(C)** Purified WT (mCD40+/+) or hCD40Tg/mCD40 KO (mCD40-/-, hCD40+/-) mouse B cells were incubated for 16 hr with anti-

mCD40 (3/23) or anti-hCD40 (ChiLob 7/4) of the indicated isotypes at 1 μg/ml. B-cell activation was assessed by homotypic adhesion (top; bar = 1mm) and CD23 upregulation (bottom; filled grey histograms, untreated cells; black line, treated with mAb alone; blue line, incubated with mAb + FcγRIIB over-expressing cross-linking cells). **(D)** Splenic sections from mice administered 100 μg of the indicated 3/23 isotypes stained for CD70 (green, left) and the DC marker MIDC8 (red, middle; merge on right). Bar = 100 μm. Results from 1 of 2 experiments shown.

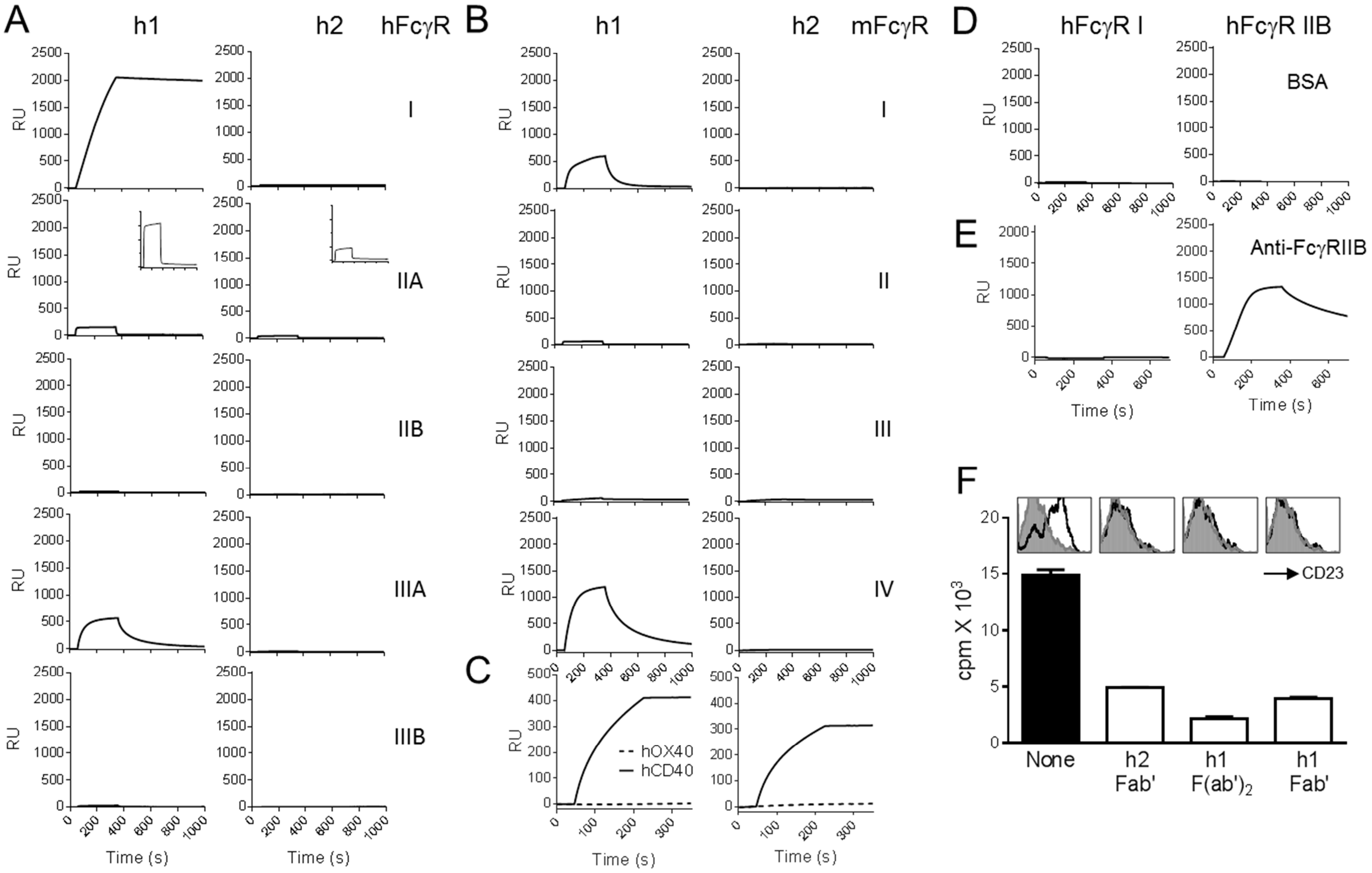

Figure S2. Related to Figure 2. ChiLob 7/4 h2 agonistic activity is FcγR independent. (A and B) SPR profiles to show binding of the indicated human (A) and mouse (B) FcγR at 100 nM to ChiLob 7/4 h1 or h2 immobilised at 15,000 RU. All profiles are presented on the same scale (Y axis to 2500 RU) to allow comparison of relative binding. The insets for FcγRIIA show the same data plotted with a Y axis scale of 200 RU to reveal low level binding. (C) To demonstrate integrity of the bound mAb, the binding of 100 nM hCD40 protein (solid line) or hOX40 protein (broken line) to the h1 and h2 mAb immobilised on the flow cells used in A and B was determined. (D) Background binding of hFcγRI and IIB to immobilised BSA. Similar results were obtained for all FcγR. This background was subtracted from the profiles in A and B. (E) To demonstrate integrity of the hFcγRIIB, anti-FcγRIIB-specific mAb (KB61) was immobilised and the binding of hFcγRI and IIB compared. (F) Purified hCD40Tg mouse B cells were incubated for 16 hours with 1 μg/ml ChiLob 7/4 h2 IgG alone (left, black bar) or in the presence of a 50-fold

excess of the indicated ChiLob 7/4 IgG fragments. CD23 expression was analysed by flow cytometry after 20 hr (top panel; grey histogram untreated cells, black line mAb treated cells) and B cell proliferation by ^{3}H thymidine incorporation after 5 days (bottom panel, mean and range of duplicate samples). Results from 1 of 2 experiments shown.

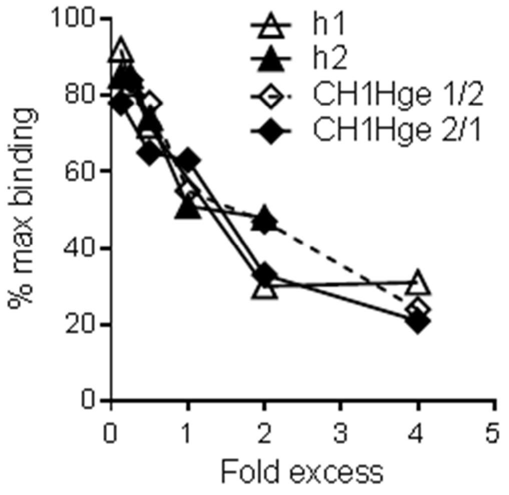

Figure S3. Related to Figure 4. ChiLob 7/4 switch mutants bind similarly to CD40.

Purified hCD40Tg mouse B cells were incubated with ChiLob 7/4 h1-FITC pre-mixed with different ratios of the indicated unlabelled mutants and analysed as in Figure S1A.

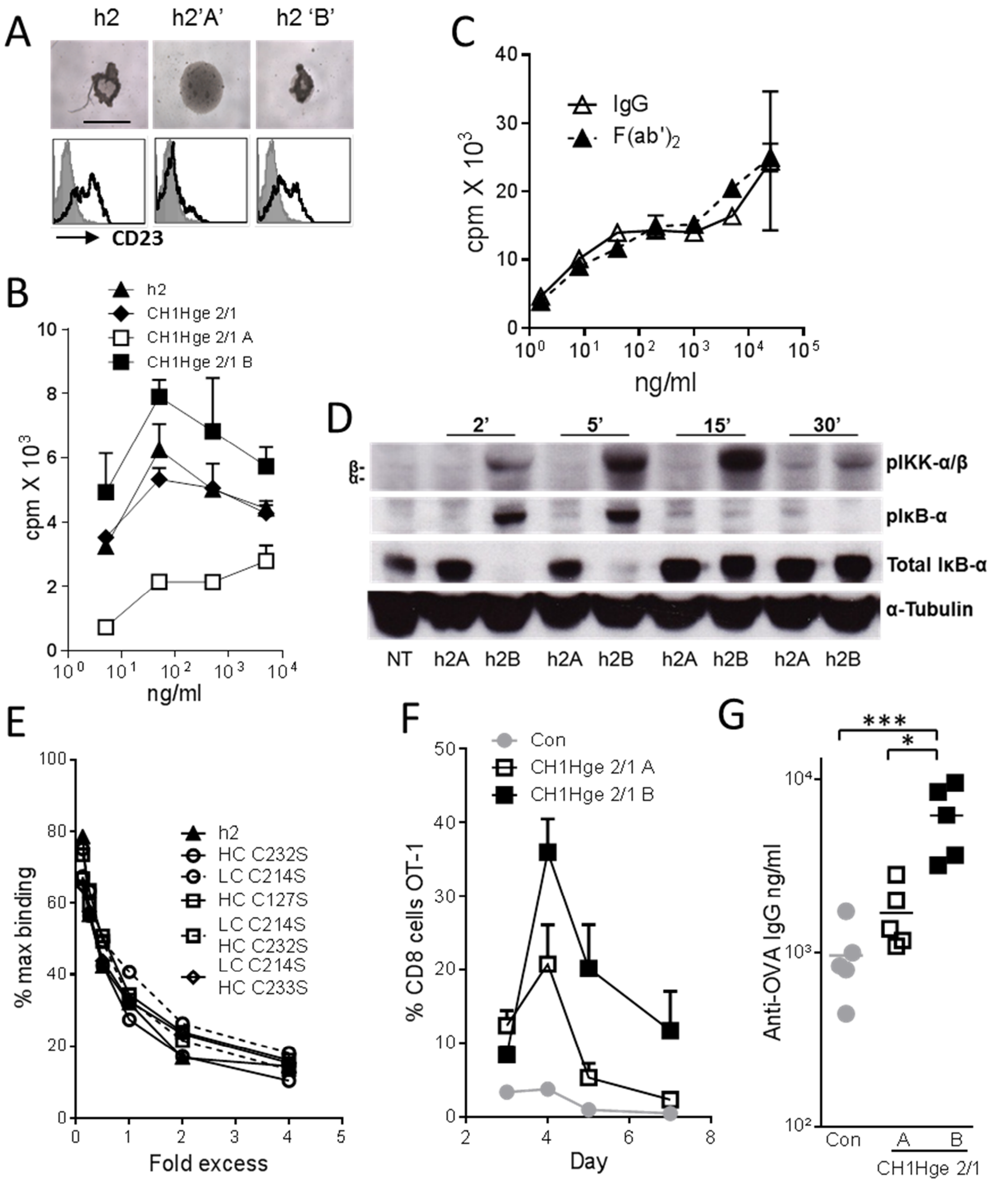

Figure S4. Related to Figure 6. Differential activity of ChiLob 7/4 h2A and h2B forms. (A) Activation of purified human B cells assessed by homotypic adhesion (top; bar = 1mm) and CD23 upregulation (bottom) after incubation with native, 'A' or 'B' skewed forms of ChiLob 7/4 h2 at 200 ng/ml for 16 hr. (B) Purified hCD40Tg B cells were incubated with the indicated concentrations of ChiLob 7/4 h2, CH1Hge 2/1 mutant or skewed forms of the mutant and proliferation measured as in Figure 6A (mean and range of duplicate samples). (C) hCD40Tg

mouse B cell proliferation in response to increasing concentrations of h2B skewed ChiLob 7/4 h2 IgG or $F(ab')_{2,}$ measured as in B. (D) Western blot of lysates from Ramos cells treated with ChiLob 7/4 h2A and h2B at 1 μg/ml for the indicated times and probed with Ab specific for phosph-IKKα/β, phospho IκB–α or IκB–α. Anti-tubulin was used as a loading control. (E) Purified hCD40Tg mouse B cells were incubated with ChiLob 7/4 h1-FITC pre-mixed with different concentrations of the indicated unlabelled mutants and analysed as in Figure S1D. (F) and (G) hCD40Tg FcγRIIB KO mice that had been adoptively transferred with OTI cells were immunised with 100 μg OVA plus 100 μg of the skewed mutant mAb as in Figure 1. Circulating OTI cells were enumerated over time (mean +/- SD for 5 animals per group) (D) and anti-OVA antibodies in the sera measured on day 18 (E). One of 2 similar experiments shown. *$p<0.05$, ***$p<0.001$.

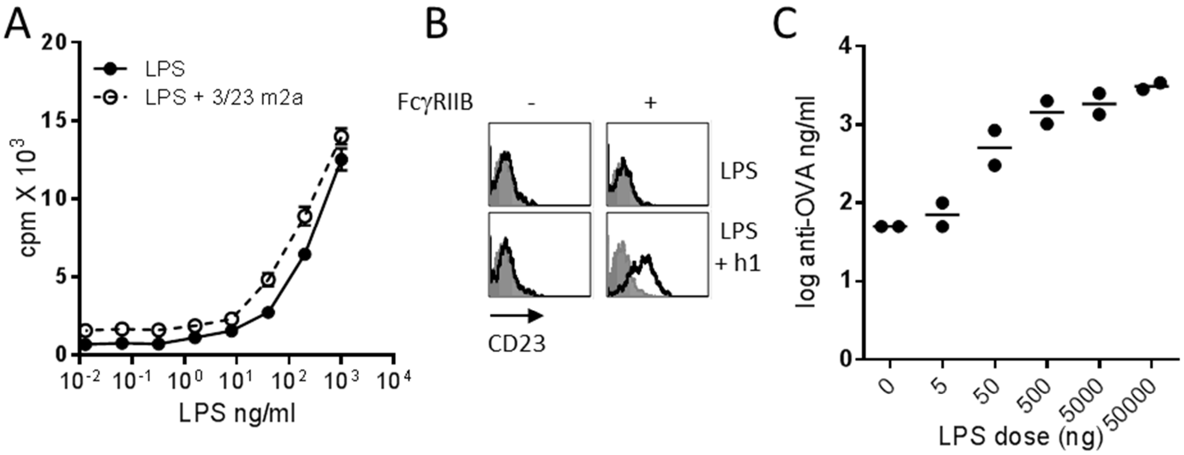

Figure S5. Related to Figure 1. LPS contamination does not account for anti-CD40 mAb activity.

(A) FcγRIIB$^{-/-}$ mouse B cells were incubated with increasing concentrations of LPS in the presence or absence of 400 ng/ml 3/23 m2a. Proliferation was assessed by ^{3}H thymidine incorporation (mean +/- SEM triplicates). (B) Human B-cell activation assessed by CD23 upregulation (black line compared to control, grey) after 16 hr incubation with 1 μg/ml LPS in the presence or absence of 1 μg/ml ChiLob 7/4 h1 and/or FcγRIIB over-expressing cross-linking cells as indicated. (C) Mice were immunised with 100 μg endotoxin-free OVA plus the indicated dose of LPS. Circulating anti-OVA Ab titres were determined on day 14.

SUPPLEMENTAL EXPERIMENTAL PROCEDURES

Chimeric antibody production and quality control

Variable regions were subcloned into expression vectors (pEE6.4 vector for heavy chain and pEE12.4 vector for light chain, Lonza) containing constant regions of different antibody isotypes. Heavy and light chain vectors were further subcloned together before transfection into 293F cells for transient or CHO-K1 cells for stable production of mAbs. Secreted mAb was purified by Protein A-Sepharose (Sigma-Aldrich) chromatography and aggregates (as revealed by SEC-HPLC) removed by gel filtration through Sephadex 200 (Sigma-Aldrich). All preparations were endotoxin low (<1 ng/mg protein) as determined by an Endosafe-PTS portable test system (Charles River Laboratories). Contaminating endotoxin could not account for the mAb functions described in this study as i) mAb concentrations of >10mg/ml would be required to provide enough endotoxin to cause mouse B cell proliferation in vitro (Figure S5A); ii) human B cells do not respond to endotoxin in vitro (Bourke et al., 2003) and Figure S5B), but do show isotype dependent differences in activation with ChiLob 7/4; iii) a dose of at least 50mg of mAb (500-fold that given) would be required to provide enough endotoxin to boost immune responses to this level in vivo (Figure S5C). Flow cytometry and/or SPR were used to assess differences in Ag binding. Non-reducing denaturing capillary electrophoresis (nrCE-SDS) of mAb preparations was performed using a Beckman PA800 Plus analyser according to the manufacturer's instructions. To produce skewed forms of h2, mAb were dialysed into 0.2 M Tris pH8.0 containing 6 mM cysteine plus 1 mM cystamine with (for h2A) or without (for h2B) 2 M guanidine hydrochloride, for 4 days at 4^0C, then dialysed into PBS before use. Pepsin digestion was used to make $(Fab')_2$ fragments that were then chemically reduced to produce Fab' as described (Glennie et al., 1987). Protein A chromatography was used to remove any residual Fc.

Cell isolation, activation and proliferation

Dendritic cells: Human primary Langerhans cells were isolated as described previously (Polak et al., 2012). Briefly, skin specimens were acquired from healthy individuals and epidermal sheets were separated following 20 hr enzymatic digestion (Disopase, 2 IU, Gibco, UK). LCs were harvested following 48 hr migration from epidermal sheets, and enriched to >70% CD1a+ HLADR+ cells by Optiprep™ density gradient (Axis Shield, Norway). Cells were plated into 96 well U-bottom plates at $5x10^4$ cells/well in RPMI 1640 (Gibco, UK) supplemented with Penicillin/Streptomycin (1%, Sigma, UK) and FBS (10%, Invitrogen, UK) and stimulated with ChiLob 7/4 human IgG1 or human IgG2 mAbs or isotype control for 18 h. Expression of activation markers CD40, CD86, CD70 on CD1a+ HLADR+ (all BD Biosciences) LC was assessed by flow cytometry.

B cells: B cells were purified from spleen (mouse) or peripheral blood mononuclear cells (PBMC, human) using magnetic negative selection kits (Miltenyi Biotech or StemCell Technologies). Human PBMC (Lymphoprep, Axis-Shield) were isolated from blood cones obtained from anonymous healthy donors through the National Blood Service (Southampton General Hospital). Cells were plated into 96-well round-bottom dishes at $1x10^5$ cells/well with various concentrations of mAb as described for individual experiments. In some cases, $1x10^5$ 293F cells transfected with human FcγR (White et al., 2011) were also added. To assess activation, cells were photographed (Olympus CKX41 microscope with CC12 soft imaging system) after overnight incubation and activation marker expression analysed by flow cytometry (FACSCalibur, BD Biosciences). Proliferation was assessed by [methyl-^{3}H] thymidine

(PerkinElmer, Cambridge, UK) incorporation after 5 (mouse) or 8 (human) days of culture, as described (White et al., 2011).

T cells: Human PBMCs were labelled with 2 μM CFSE and then pre-cultured for 2 days at high density in 24-well plates as described (Romer et al., 2011), with 1.5 ml of cells at 1 x 10^7 /ml per well. Pre-cultured cells were washed and resuspended at 1 x 10^6/ml for the assay. For some experiments T cells were isolated from pre-cultured PBMCs using a total T-cell isolation kit (Miltenyi Biotec). For the anti-h4-1BB mAbs, wells of 96-well round- bottomed plates were coated with 0.02 μg/ml OKT3 in PBS for 4 h, then washed twice and 10^5 PBMCs /well incubated with 5 μg/ml mAb (final volume 150 μl) for 5 days. Proliferation of $CD4^+$ cells was assessed by flow cytometric analysis of CFSE dilution. For the anti-CD28 mAbs, 10^5 isolated T cells were incubated with mAb in uncoated wells and proliferation assessed as above. Results are expressed as the percentage of divided cells.

For activation of EBV-peptide specific CD8+ human T lymphocytes; HLA-A2 restricted T cells specific for the BMLF-1 epitope of EBV (GLCTLVAML; Cambridge Peptides, UK) were expanded from HLA-A2 individuals as described (Polak et al., 2012). Human primary Langerhans cells (LCs) were incubated with an extended long peptide containing BMLF-1 (proGLC: FNNFTVSFWLRVPKVSASHLEGLCTLVAML,10 μM) for 6h and stimulated with ChiLob 7/4 h1 or h2 mAb or isotype control, 100 ng/ml for 18 h. Pulsed and washed LCs (1x10^4 cells) were co-cultured with BMLF-1-specific T cells (5x10^4 cells) for 20 hours in an ELISpot assay for IFN-γ production (Mabtech, Sweden) as per manufacturer's protocol. Spot forming units (sfu) were enumerated with ELISpot 3.5 reader.

Surface Plasmon Resonance.

A Biacore T100 (GE Healthcare) was used to compare the relative interactions between soluble Fcγ receptors and ChiLob7/4 mAb isotypes. Antibodies or BSA as a reference were immobilised at 15,000 RU to CM5 sensor chips (Biacore) by standard amine coupling according to the manufacturer's instructions. Use of an isotype control mAb to coat the reference flow cell was ruled out due to the presence of Fc. Soluble FcγR (R and D Systems, Abingdon, UK) were injected through the flow cell at 100 nM in HBS-EP+ running buffer (Biacore) at a flow rate of 30 µl/min at 25^{0}C. Regeneration was performed for 30 seconds with 10mM glycine, pH 2. The integrity of the mAb coated onto the flow cells was checked by using positive (hCD40-Fc) and negative (hOX40-Fc) control fusion proteins (R and D systems) at 100 nM (Figure S2C). The background response for the reference flow cell was subtracted automatically; binding to the reference cell was negligible for all FcγR (Figure S2D and data not shown). The integrity of each of the purified FcγR proteins was confirmed by at least one of the following: expected binding profiles for IgG isotypes (White et al., 2011) and this study); increased binding to mAb with mutated Fc known to enhance FcγR interaction (not shown); binding by immobilised anti-FcγR mAb specific for individual FcγR (not shown and Figure S2E). Conditions for the comparison of the interaction between the different anti-CD40 mAb and immobilised hCD40 were as follows: for comparison of Fab' binding, hCD40-Fc (R and D Systems) was immobilised at pH5 at 1000 RU as above and Fab' fragments passed over at 640, 128, 25.6, 5.12 and 1.024 nM (Figure S1E); for comparison of IgG binding, hCD40-Fc was immobilised at 8000 RU and IgG passed over at 100, 20, 4, 0.8 and 0.16 nM (Figure 6F). Regeneration was performed for 30 seconds with 10mM glycine, pH 1.5. Affinities of ChiLob 7/4 h1 and h2 Fab' fragments for CD40 were determined using Biacore Evaluation Software fitting a 1:1 binding model.

SUPPLEMENTAL REFERENCES

Bourke, E., Bosisio, D., Golay, J., Polentarutti, N., and Mantovani, A. (2003). The toll-like receptor repertoire of human B lymphocytes: inducible and selective expression of TLR9 and TLR10 in normal and transformed cells. Blood *102*, 956-963.

Glennie, M. J., McBride, H. M., Worth, A. T., and Stevenson, G. T. (1987). Preparation and performance of bispecific F(ab' gamma)2 antibody containing thioether-linked Fab' gamma fragments. J Immunol *139*, 2367-2375.

Polak, M. E., Newell, L., Taraban, V. Y., Pickard, C., Healy, E., Friedmann, P. S., Al-Shamkhani, A., and Ardern-Jones, M. R. (2012). CD70-CD27 interaction augments CD8+ T-cell activation by human epidermal Langerhans cells. J Invest Dermatol *132*, 1636-1644.

Romer, P. S., Berr, S., Avota, E., Na, S. Y., Battaglia, M., ten Berge, I., Einsele, H., and Hunig, T. (2011). Preculture of PBMCs at high cell density increases sensitivity of T-cell responses, revealing cytokine release by CD28 superagonist TGN1412. Blood *118*, 6772-6782.
