# Supplementary material for: Conformation of the Human Immunoglobulin G2 Hinge Imparts Superagonistic Properties to Immunostimulatory Anticancer Antibodies
Source: Cancer Cell. 2015 Jan 12;27(1):138–48. doi: 10.1016/j.ccell.2014.11.001 (PMC4297290; doi:10.1016/j.ccell.2014.11.001)
Supplement: Document S2. Article plus Supplemental Information [file mmc2.pdf]

Cell Press

Cancer Cell
Article

Conformation of the Human Immunoglobulin G2 Hinge Imparts Superagonistic Properties to Immunostimulatory Anticancer Antibodies

Ann L. White,[1,*] H.T. Claude Chan,[1] Ruth R. French,[1] Jane Willoughby,[1] C. Ian Mockridge,[1] Ali Roghanian,[1] Christine A. Penfold,[1] Steven G. Booth,[1] Ali Dodhy,[1] Marta E. Polak,[2] Elizabeth A. Potter,[1] Michael R. Ardern-Jones,[2] J. Sjef Verbeek,[3] Peter W.M. Johnson,[1] Aymen Al-Shamkhani,[1] Mark S. Cragg,[1] Stephen A. Beers,[1] and Martin J. Glennie[1]

[1]Cancer Sciences Unit, Faculty of Medicine, University of Southampton, Tremona Road, Southampton SO16 6YD, UK

[2]Clinical and Experimental Sciences, Faculty of Medicine, University of Southampton, Tremona Road, Southampton SO16 6YD, UK

[3]Department of Human Genetics, Leiden University Medical Centre, Albinusdreef 2, 2333 ZA Leiden, the Netherlands

*Correspondence: a.l.white@soton.ac.uk

http://dx.doi.org/10.1016/j.ccell.2014.11.001

This is an open access article under the CC BY license (http://creativecommons.org/licenses/by/3.0/).

SUMMARY

Monoclonal antibody (mAb) drugs that stimulate antitumor immunity are transforming cancer treatment but require optimization for maximum clinical impact. Here, we show that, unlike other immunoglobulin isotypes, human IgG2 (h2) imparts FcγR-independent agonistic activity to immune-stimulatory mAbs such as anti-CD40, -4-1BB, and -CD28. Activity is provided by a subfraction of h2, h2B, that is structurally constrained due its unique arrangement of hinge region disulfide bonds. Agonistic activity can be transferred from h2 to h1 by swapping their hinge and CH1 domains, and substitution of key hinge and CH1 cysteines generates homogenous h2 variants with distinct agonistic properties. This provides the exciting opportunity to engineer clinical reagents with defined therapeutic activity regardless of FcγR expression levels in the local microenvironment.

INTRODUCTION

Monoclonal antibodies (mAbs) that modulate immune responses are proving highly effective in cancer treatment, with increasing evidence that such responses can be harnessed to provide durable eradication of tumors (Hodi et al., 2010; Sliwkowski and Mellman, 2013; Topalian et al., 2012; Wolchok et al., 2013). Results with "checkpoint blocker" mAbs designed to antagonize the inhibitory T cell coreceptors cytotoxic T lymphocyte antigen 4 (CTLA-4) and programmed death 1 have reinforced the view that T cell immunity can provide long-lasting protection against aggressive and difficult-to-treat cancers, such as metastatic melanoma and non-small-cell lung cancer (Hodi et al., 2010; Topalian et al., 2012; Wolchok et al., 2013). Promising clinical data are also emerging with immunostimulatory mAbs that bind agonistically to the costimulatory receptor CD40 on antigen-presenting cells (APCs) (Beatty et al., 2011, 2013; Vonderheide and Glennie, 2013) with agents against a number of other costimulatory targets, such as 4-1BB (CD137), OX40 (CD134), and glucocorticoid-induced tumor necrosis factor receptor-related protein (GITR), in clinical development (Moran et al., 2013). These agonistic agents also have the potential to enhance therapeutic efficacy of other anticancer mAbs, such as those directed against CD20 or epidermal growth factor receptor (EGFR). As demonstrated by Levy and colleagues, stimulation of 4-1BB on natural killer (NK) cells promotes their cytotoxic potential and enhances antibody (Ab)-dependent cell-mediated cytotoxicity (ADCC) of anti-CD20-, anti-EGFR-, or anti-human epidermal growth factor receptor 2 (HER2)-coated tumor cells (Kohrt et al., 2011, 2012, 2014). Despite clinical success, however, only a minority of patients show durable responses to immunomodulatory agents, and a detailed understanding of

Significance

Monoclonal antibodies (mAbs) that stimulate anticancer immunity provide curative therapy in a subset of patients with traditionally terminal malignancies. Realization of the full potential of these agents, however, will require precise engineering provided by a detailed understanding of their mechanisms of action. Here, we demonstrate that human IgG2 (h2) constant regions provide mAbs targeting three immunostimulatory coreceptors in clinical development—CD40, 4-1BB, and CD28—with agonistic activity independent of Fcγ receptor interaction that is usually required for receptor clustering and downstream intracellular signaling. This exceptional activity is conferred by the unique configuration of disulfide bonds in the h2 hinge and paves the way for engineering improved clinical reagents with defined activity regardless of FcγR expression in the local microenvironment.

their mechanisms of action remains unclear, making it difficult to rationally optimize therapeutic activity.

One factor that has a crucial impact on therapeutic efficacy is mAb isotype due largely to differences in Fcγ receptor (FcγR) interactions that influence events downstream of antigen engagement (Nimmerjahn and Ravetch, 2012; White et al., 2013). Direct targeting anticancer mAbs, such as anti-CD20, -EGFR, and -HER2, work at least in part by deletion of their cellular targets through ADCC and as such require interaction with activatory FcγR on NK cells and macrophages (Clynes et al., 2000; Kurai et al., 2007; Uchida et al., 2004). Mouse immunoglobulin G (IgG) 2a and human IgG1 (h1) are effective for this type of agent as they preferentially engage activatory rather than inhibitory FcγR (Hamaguchi et al., 2006; Nimmerjahn and Ravetch, 2005). Recent studies in preclinical models have shown a similar isotype dependence for some immunomodulatory mAbs (anti-CTLA-4, -GITR, and -OX40) where depletion of target-expressing T regulatory cells in the tumor is demonstrated to be at least partly responsible for therapeutic efficacy (Bulliard et al., 2013, 2014; Simpson et al., 2013). In contrast, mAbs whose effects rely on agonistic receptor engagement, such as anti-CD40 (Li and Ravetch, 2011; White et al., 2011, 2014) or apoptosis-promoting anti-death receptor (DR) 4, DR5, and Fas (Li and Ravetch, 2012; Wilson et al., 2011; Xu et al., 2003), appear to rely predominantly on crosslinking by the inhibitory FcγRIIB to deliver their activity (Li and Ravetch, 2013; White et al., 2011, 2014). For this type of agent, mouse IgG1 (m1) is optimal in preclinical models as it binds with sufficient affinity to FcγRIIB to mediate crosslinking (Li and Ravetch, 2011; White et al., 2011, 2014). A similar mechanism appears to be required for human mAbs as, although human IgG isotypes bind with low affinity to FcγRIIB as determined by surface plasmon resonance (SPR; Bruhns et al., 2009), enhancing the affinity of human IgG1 to human FcγRIIB through Fc engineering is effective in bestowing agonistic activity on non-agonist anti-CD40 mAbs both in vitro (White et al., 2013) and in mice overexpressing human FcγRIIB (Li and Ravetch, 2011; Li and Ravetch, 2012). In addition, Bartholomaeus et al. (2014) show that FcγRIIB-mediated crosslinking is required to deliver agonist activity to the human IgG4 (h4) anti-human CD28 mAb, TGN1412, in vitro. This study elegantly confirms that at the cell-cell interface, when multiple Fc regions of immobilized IgG may be engaged by FcγRIIB, the affinity of human IgG for this receptor binding may be both sufficient and necessary to promote agonistic activity (Lux et al., 2013). However, agents that rely on FcγRIIB-mediated crosslinking will always be limited by the availability of the receptor. An alternative is to develop superagonistic reagents that promote immune responses without FcγR engagement (White et al., 2014).

To investigate this latter option, we undertook an evaluation of the immunomodulatory activity of human IgG isotypes using mAbs against a number of human costimulatory receptors (CD40, 4-1BB, and CD28) currently being investigated as therapeutic targets. We show that h2 imparts greater immunostimulatory activity to these agents than either h1 or h4. Detailed studies with anti-CD40 demonstrated that this activity did not depend upon differences in FcγR interaction, but rather on the unique configuration of disulfide bonds in the h2 hinge and CH1 domains. Furthermore, using genetic engineering, we could manipulate these bonds and "lock" the mAbs into different configurations with contrasting levels of agonistic activity. This strategy provides the opportunity to develop homogeneous superagonistic therapeutic agents with defined levels of activity that are independent of FcγR expression levels in the local microenvironment.

RESULTS

Human IgG Isotypes and Anti-CD40 Activity

To examine the effect of human constant regions on agonistic activity, we used the anti-human CD40 mAb ChiLob 7/4 (Chowdhury et al., 2014), currently in phase 1 clinical trial in cancer patients. Agonistic activity was initially assessed by the ability of ChiLob 7/4 to cause human B cell activation and proliferation in vitro when chimerized onto different human constant regions. All chimeras bound similarly to CD40 as determined by flow cytometry (Figure S1A available online). Fab′ fragments of ChiLob 7/4 h1 and ChiLob 7/4 h2 also bound to immobilized hCD40 with similar affinities (10.0 and 10.2 × 10^{-9} M, respectively) as measured by SPR (Figure S1B). When added to purified human B cells, ChiLob 7/4 h2 caused much greater B cell activation and proliferation as assessed by homotypic adhesion (cell clumping) and [^{3}H]thymidine uptake, respectively, compared to ChiLob 7/4 h1 or ChiLob 7/4 h4 (Figure 1A). To determine that Fab′ arm exchange did not account for the lack of activity of h4, we introduced a stabilizing S228P mutation into ChiLob 7/4 h4 (Angal et al., 1993). This alteration (h4* in Figure 1A) did not alter activity.

In preclinical models immune stimulatory and therapeutic effects of anti-CD40 mAb can be mediated through upregulation of the costimulatory molecule CD70 on dendritic cells (DCs), resulting in enhanced CD8 T cell immunity (French et al., 1999, 2007; Sanchez et al., 2007). To compare the ability of ChiLob 7/4 h1 and ChiLob 7/4 h2 to activate human DCs, we assessed their capacity to upregulate CD70 expression on primary human Langerhans cells in vitro (Figure 1B), as well as their ability to enhance Langerhans cell-induced priming of Epstein-Barr virus-specific human CD8 T cells measured by interferon γ (IFN-γ) production (Figure 1C). Consistent with effects on B cells, ChiLob 7/4 h2 enhanced both Langerhans cell CD70 expression and CD8 T cell priming, whereas ChiLob 7/4 h1 did not.

The in vivo agonistic activity of ChiLob 7/4 isotypes was examined in hCD40 transgenic (Tg) mice (Ahonen et al., 2002). Initial in vitro studies confirmed that hCD40 in mouse cells responded to ligation with ChiLob 7/4 similarly to that in human cells, as ChiLob 7/4 h2 stimulated much greater activation and proliferation of isolated hCD40 Tg mouse B cells than ChiLob 7/4 h1 (Figure 1D) that was dependent upon the expression of hCD40 (Figure S1C). To test in vivo agonistic activity, we examined the ability of ChiLob 7/4 to enhance CD8 T cell and Ab responses when coadministered with the model antigen ovalbumin (OVA). Consistent with the in vitro data, ChiLob 7/4 h2 stimulated significantly greater anti-OVA CD8 T cell expansion as well as Ab responses than observed with ChiLob 7/4 h1 (Figure 1E). Thus, using a series of both in vitro and in vivo approaches, ChiLob 7/4 demonstrated greater immunostimulatory activity when expressed with h2 versus h1 or h4 constant regions.

Similar differences between h1 and h2 agonistic activity were also observed in vivo when we examined the anti-mouse CD40 mAb 3/23, where again 3/23 h2 but not 3/23 h1 stimulated potent anti-OVA CD8 T cell and Ab responses (Figure 1F) and

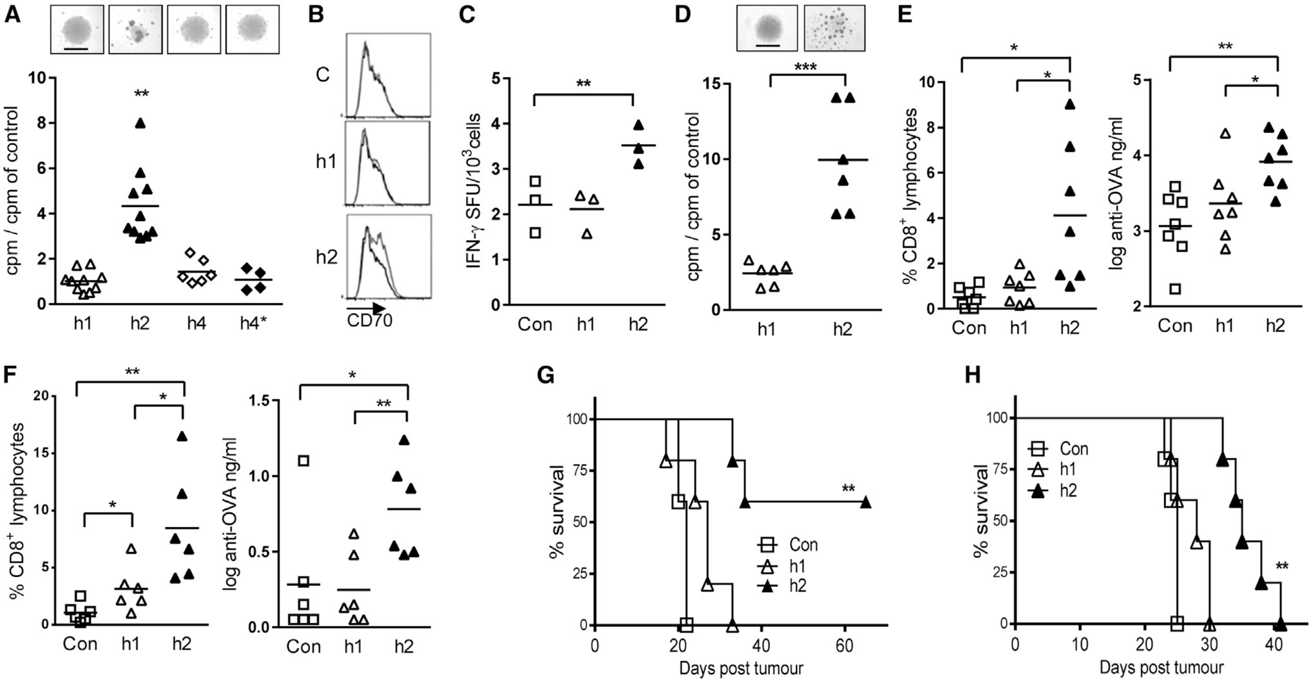

Figure 1. Human Isotypes and Anti-CD40 Activity

(A) Activation of human B cells in response to ChiLob 7/4 of the indicated isotypes (1 μg/ml) was assessed after 16 hr by homotypic adhesion (top; bar, 1 mm) and [^{3}H]thymidine incorporation. Points represent individual samples from two to five experiments per isotype.

(B) Human Langerhans cells were untreated (black line) or incubated (gray line) with ChiLob 7/4 h1, ChiLob 7/4 h2, or isotype control (C) for 18 hr, and CD70 expression was analyzed by flow cytometry; one of two experiments shown.

(C) IFN-γ ELISpot assay of BMLF-1-specific CD8+ T cell activation by human leukocyte antigen-matched human Langerhans cells activated with ChiLob 7/4 as in (B). Data are representative of two experiments in triplicate normalized to activation by unpulsed cells.

(D) Activation of hCD40 Tg B cells by ChiLob 7/4 h1 and ChiLob 7/4 h2 at 200 ng/ml was analyzed as in (A). Points are individual samples from three experiments performed in duplicate.

(E) hCD40 Tg mice were immunized with 100 μg of OVA with or without (Con) 100 μg of the indicated ChiLob 7/4 mAb. Circulating endogenous OVA-specific CD8+ T cells were enumerated day 8, and the peak of the response (left) and anti-OVA Ab (right) was determined on day 14. Individual animals from two of four experiments shown.

(F) Mice (n = 3) adoptively transferred with OTI cells were immunized with 100 μg of endotoxin-free OVA without (Con) or with 100 μg of the indicated 3/23 mAb. Circulating OTI cells were enumerated at the peak of the response (day 5; left) and anti-OVA Ab at day 14 (right). Combined data from two of more than five experiments.

(G) C57Bl/6 mice (n = 5) were adoptively transferred with OTI cells, immunized with OVA plus 100 μg of the indicated 3/23 isotypes, and 5 days later were challenged with EG7 tumor subcutaneously. Survival curves from one of two experiments shown.

(H) BALB/c mice (n = 5) were challenged intravenously (i.v.) with BCL_1 tumor cells and 14 days later (when the tumor represented 5%–10% spleen weight [White et al., 2014]) were given i.v. 100 μg of 3/23 h1, 3/23 h2, or PBS (Con) as indicated. Survival curves from one of two experiments shown. *$p < 0.05$, **$p < 0.01$, ***$p < 0.001$.

See also Figure S1.

upregulated CD70 on splenic DCs (Figure S1D). Importantly, this difference in immunostimulatory capacity correlated with differences in therapeutic activity where 3/23 h2, but not 3/23 h1, provided protection against tumor development in both a vaccination setting where mice immunized with OVA plus anti-CD40 were challenged with the OVA-expressing EG7 tumor (Figure 1G) and a therapeutic setting where mice with established B cell lymphoma (BCL_1 lymphoma model [White et al., 2014]; Figure 1H) were treated with a single 100 μg dose of mAb.

Human IgG2 Activity Is FcγR Independent

The low affinity of h2 for FcγRIIB (Bruhns et al., 2009), the predominant FcγR on B cells, suggested that, unlike agonistic m1 that uses FcγRIIB as a crosslinker (White et al., 2011), its activity may be FcγR independent. Indeed, SPR where soluble FcγRs were passed over immobilized mAb revealed very little binding of ChiLob 7/4 h2 to any human or mouse FcγR (Figures S2A and S2B), whereas ChiLob 7/4 h1 clearly bound hFcγRI, -IIA, and -IIIA as well as mFcγRI and -IV under the same conditions (Figures S2A and S2B). Control experiments confirmed the integrity of the mAb and FcγR proteins used (Figures S2C–S2E). A number of approaches confirmed the FcγR-independent activity of h2. First, a pan-blocking anti-FcγRII mAb (AT10) failed to prevent activation of human B cells by ChiLob 7/4 h2, as assessed by homotypic adhesion and CD23 upregulation (Figure 2A). In contrast, AT10 completely blocked activation by ChiLob 7/4 h1 induced in the presence of FcγRII-expressing crosslinking cells (Figure 2A). Second, removal of the ChiLob 7/4 h2 Fc through pepsin cleavage (producing F(ab′)$_2$ fragments) did not prevent activation and proliferation of human B cells,

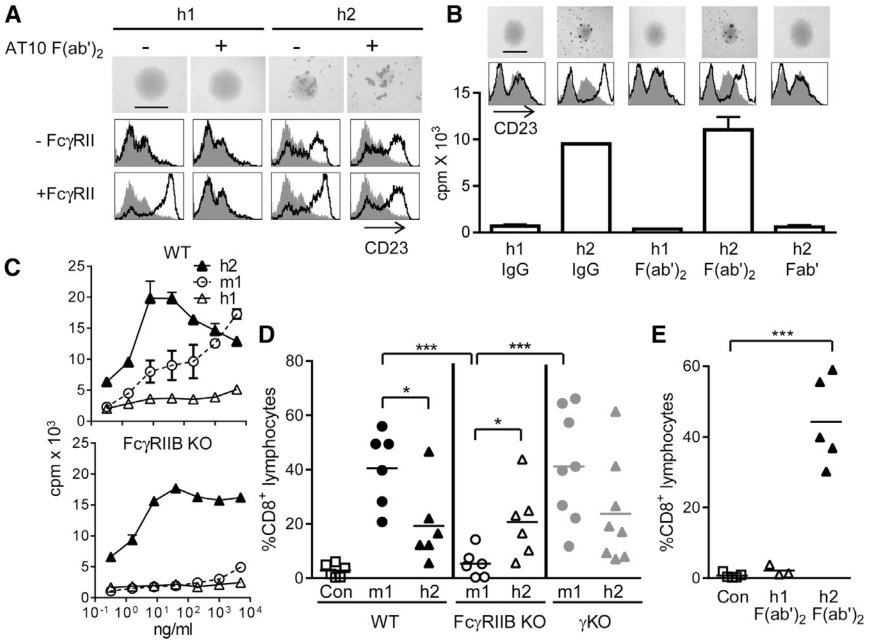

Figure 2. FcγR-Independent Activity of Human IgG2

(A) Activation of human B cells by ChiLob 7/4 h1 or ChiLob 7/4 h2 plus or minus a 50-fold excess of blocking anti-FcγRII (AT10) $F(ab')_2$ and/or hFcγRIIB-overexpressing 293F cells (+/− FcγRII) as indicated, assessed by homotypic adhesion (top; bar, 1 mm) and CD23 expression (treated cells, black line; unstimulated cells, gray histogram).

(B) Activation of human B cells by ChiLob 7/4 h1 and ChiLob 7/4 h2 whole IgG, $F(ab')_2$, or Fab′ for 16 hr at 1 μg/ml assessed by homotypic adhesion (top; bar, 1 mm), CD23 upregulation (middle), and proliferation (bottom). Means and ranges of duplicate samples from one of four experiments.

(C) Proliferation of hCD40 Tg B cells WT or KO for FcγRIIB with various concentrations of the indicated ChiLob 7/4 isotypes determined by [^{3}H] thymidine incorporation (mean and range of duplicates, one of four experiments).

(D) hCD40 Tg mice (n = 3–5) that were FcγR WT, FcγRIIB KO, or common γ chain KO (no activatory FcγR) received OTI cells and then OVA plus the indicated ChiLob 7/4 mAb. Circulating OTI cells were enumerated at day 5. Combined results from two experiments.

(E) hCD40Tg/FcγRIIB KO mice received OTI cells then were immunized with OVA alone (Con) or with 200 μg of ChiLob 7/4 h1 Fab'_2 or ChiLob 7/4 h2 Fab'_2 i.v. on day 0 followed by 100 μg of Fab'_2 on days 1 and 2. Circulating OTI cells on day 5 are shown. Similar results were obtained when mice were given a single 100 μg dose of h2 Fab'_2 i.v. (not shown). ***$p < 0.001$, *$p < 0.05$.

See also Figure S2.

whereas reduction to Fab′ eliminated activity (Figure 2B). In contrast, under the same conditions ChiLob 7/4 h1 was unable to activate cells when added as IgG, $F(ab')_2$, or Fab′, although when added in excess, each form prevented activation by ChiLob 7/4 h2 (Figures 2B and S2F). Third, genetic deletion of FcγRIIB from mouse B cells, the only FcγR expressed by these cells, did not prevent their proliferation in response to ChiLob 7/4 h2 over a wide concentration range (Figure 2C), whereas response to ChiLob 7/4 m1, that is dependent on FcγRIIB cross-linking for activity (White et al., 2011), was lost (Figure 2C). Of note, ChiLob 7/4 h2 produced a characteristic bell-shaped response curve when used to stimulate B cells at different concentrations and was active at very low levels in contrast to cross-linking-dependent ChiLob 7/4 m1 whose activity increased as concentrations rose (Figure 2D). These different curves presumably reflect the different mechanisms by which the isotypes impart agonistic activity and are discussed further below.

The FcγR-independent activity of ChiLob 7/4 h2 was also confirmed in vivo. Genetic deletion of FcγRIIB, previously shown to result in loss of agonistic activity of mAb against CD40 (Li and Ravetch, 2011; White et al., 2011, 2014), Fas, DR4, and DR5 (Li and Ravetch, 2012; Wilson et al., 2011; Xu et al., 2003) did not reduce expansion of OVA-specific CD8 T cells induced by ChiLob 7/4 h2 compared to that observed in wild-type (WT) mice, whereas, as expected, activity of ChiLob 7/4 m1 was lost in the FcγRIIB knockout (KO) (Figure 2D). Both mAbs remained active in γ chain KO mice that have no activatory FcγR (Figure 2D). Finally, ChiLob 7/4 h2 provided robust stimulation of CD8 T cell responses in vivo when administered as a $F(ab')_2$ fragment, whereas no response was observed with $F(ab')_2$ of ChiLob 7/4 h1 (Figure 2E).

Human IgG2 Is Agonistic for Multiple Targets

We next evaluated the influence of h2 constant regions on the activity of another hCD40 mAb in clinical trial, SGN40, also a chimeric h1 (Advani et al., 2009). The variable regions of SGN40 were synthesized from the patent sequence and chimerized onto h1 and h2 constant regions, to produce SGN40-Soton h1 and SGN40-Soton h2. Similar to ChiLob 7/4, SGN40-Soton h2 provided greater activation and proliferation of human B cells than SGN40-Soton h1 (Figure 3A), and proliferation of hCD40 Tg B cells in response to SGN40-Soton h2, but not its parental m1, S2C6 (Hanks et al., 2005), was independent of FcγRIIB expression (Figure 3B). In addition, SGN40-Soton h2 significantly and potently increased OVA-specific CD8 T cell responses in vivo in FcγRIIB KO mice, whereas SGN40-Soton h1 did not (Figure 3C). Further studies with mAbs directed against two other costimulatory receptors in clinical development, h4-1BB and hCD28, revealed similar differences in h1 and h2 agonistic function assessed by human T cell proliferation in vitro (Figures 3D and 3E). For hCD28, purified T cells were used. T cells generally lack FcγR but displayed homotypic adhesion as well as increased proliferation in response to anti-hCD28 h2, again supporting an FcγR-independent mode of action (Figure 3E). Similar to ChiLob 7/4 (Figure 1A), h4 constant regions did not confer activity to anti-hCD28 in purified T cell cultures (Figure 3E).

Agonistic Activity Depends on Both the Human IgG2 CH1 and Hinge Regions

As the variable regions of the h1 and h2 ChiLob 7/4 mAbs were identical and the activity of h2 was independent of its Fc domain, we examined the role of the CH1 and hinge domains of ChiLob 7/4 h2 in agonistic activity. To this end we produced mutants in

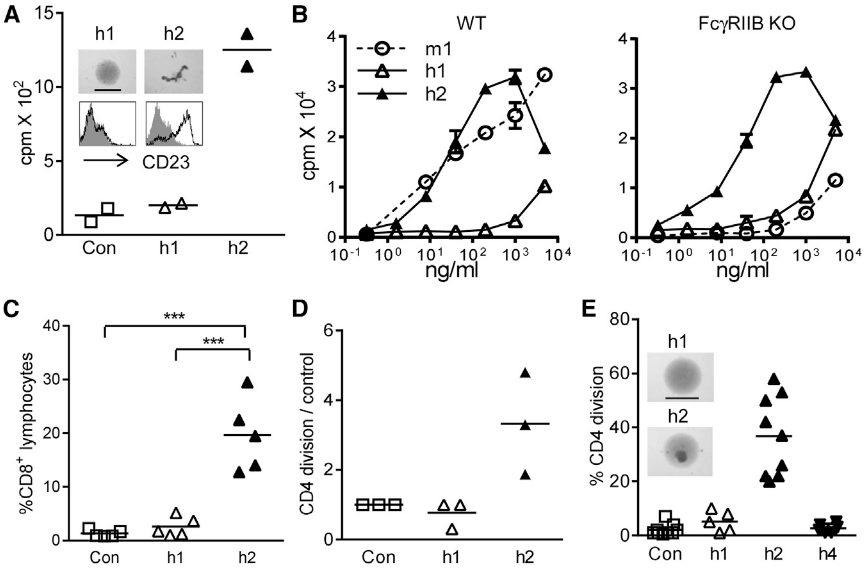

Figure 3. Human IgG2 Is Agonistic for Multiple Receptor Targets

(A) Activation of human B cells with SGN40-Soton h1 or SGN40-Soton h2 was assessed as in Figure 1A by homotypic adhesion (bar, 1 mm), CD23 upregulation (black line, treated cells; gray histogram, unstimulated controls), and proliferation (duplicate samples from one of five experiments shown).

(B) hCD40 Tg B cell proliferation (WT or FcγRIIB KO) in response to SGN40 h1 and SGN40-Soton h2 or the parental S2C6 m1 (mean and range of duplicates, one of three experiments).

(C) hCD40Tg/FcγRIIB KO mice (n = 5) received OTI cells and were then immunized with OVA alone (Con) or with 100 μg of SGN40-Soton h1 or SGN40-Soton h2. Circulating OTI cells were enumerated on D5. One of two experiments shown. ***$p < 0.001$.

(D) Proliferation of human CD4 T cells in total PBMC cultures in response to chimeric h1, h2, or h4 anti-h4-1BB.

(E) Activation assessed by homotypic adhesion (bar, 1 mm) and proliferation of purified human CD4 T cells in response to chimeric h1, h2, or h4 anti-hCD28. Points represent individual donors.

which either the CH1 domain alone or both the CH1 and hinge domains of ChiLob 7/4 h1 and ChiLob 7/4 h2 were switched (Figure 4A). Domain swapping did not interfere with antigen binding as assessed by flow cytometry (Figure S3). Comparative agonistic activity of the different mAbs was assessed by their ability to promote activation of human B cells and proliferation of hCD40 Tg B cells in vitro (Figure 4A). When either the CH1 domain of h2 was replaced with that of h1 (CH1 1/2) or the CH1 of h1 was replaced with that of h2 (CH1 2/1) (Figure 4A, i and ii), little B cell proliferation was seen and human B cells were not activated unless cells expressing high levels of FcγRIIB were provided to crosslink the mAb. Thus, the presence of either the h2 CH1 domain alone (in CH1 2/1) or the h2 hinge region alone (in CH1 1/2) did not confer activity. Similarly, no activity was seen when both the CH1 and hinge of h2 were replaced with that of h1 (CH1Hge 1/2) (Figure 4A, iii). However, when the CH1 and hinge of h1 were replaced with that of h2 (CH1Hge 2/1) (Figure 4A, iv) robust human B cell activation and proliferation of both FcγRIIB WT and KO hCD40 Tg B cells was observed, similar to that seen with native h2. Similarly, in vivo, ChiLob 7/4 CH1Hge 2/1 produced significant increases in OVA-specific CD8 T cell expansion, whereas ChiLob 7/4 CH1Hge 1/2 was inactive (Figure 4B). These data show that the unusual agonistic activity of h2 requires both its CH1 and hinge domains.

Human IgG2 Activity Is Dependent upon Its Disulfide Bond Configuration

IgG2 is unique among human IgG in its ability to "shuffle" disulfide bonds in its CH1 and hinge regions (Figure 5), resulting in a range of isoforms (Dillon et al., 2008; Martinez et al., 2008; Wypych et al., 2008; Zhang et al., 2010). The molecule is believed to be synthesized in its "h2A" form, wherein the heavy chain (HC) Cys127 in CH1 is linked to Cys214 in the light chain (LC), which then gradually converts in the blood through a series of intermediates (Liu et al., 2008) to its "h2B" form in which HC Cys127 and LC Cys214 form disulfide bonds with the HC hinge Cys232 and Cys233 (Figure 5A). Importantly, physicochemical properties (Dillon et al., 2008) and electron microscopy (Ryazantsev et al., 2013) suggest that h2A has a classical IgG flexible "Y" conformation, whereas h2B adopts a more compact shape with the Fab′ arms held in close proximity to the hinge. The h2A and h2B forms can be distinguished by non-reducing capillary electrophoresis (nrCE-SDS; Martinez et al., 2008) where they are revealed as a double peak in unfractionated h2 compared to a single peak for h1 (Figure 5B). Of the ChiLob 7/4 mAb mutants analyzed above, only CH1Hge 2/1 retained a double peak on nrCE-SDS (Figure 5B), supporting our hypothesis that disulfide shuffling is important for agonistic activity.

To determine whether differentially disulfide-linked forms of ChiLob 7/4 were associated with different agonistic activities, two approaches were taken. First, chemical "skewing" of ChiLob 7/4 in redox buffer in the presence or absence of denaturant was used to enrich for h2A or h2B, respectively (Dillon et al., 2008) (Figure 6A, top). This resulted in markedly different activities, with much greater activation of hCD40 Tg B cells with skewed h2B than h2A (Figure 6A). Similar differences in B cell activation were observed when the skewed forms were added to human B cells (Figure S4A), for skewed forms of the ChiLob 7/4 CH1Hge 2/1 mutant (Figure S4B) and for the anti-mouse CD40 mAb 3/23 where 3/23 h2B was able to activate mouse B cells in soluble form, whereas h2A required coincubation with FcγRIIB-expressing crosslinking cells (Figure 6B; these cells express non-physiologically high levels of FcγRIIB [White et al., 2011] capable of crosslinking h2). The skewed h2B form of ChiLob 7/4 was also able to activate FcγRIIB KO B cells as both whole IgG (Figure 6C, i) or as a $F(ab')_2$ fragment (Figure S4C), confirming its activity remained FcγR independent. Second, mutagenesis was used to produce "locked" h2A- and h2B-like forms as previously described (Allen et al., 2009; Martinez et al., 2008). HC C232S or C233S mutation of ChiLob 7/4 produced homogenous h2A mAbs as assessed by nrCE-SDS (Figure 6D, HC C232S, HC C233S) that did not stimulate hCD40 Tg mouse B cell proliferation at any concentration tested over a wide range (Figure 6C, ii and iii), whereas the h2B-like HC

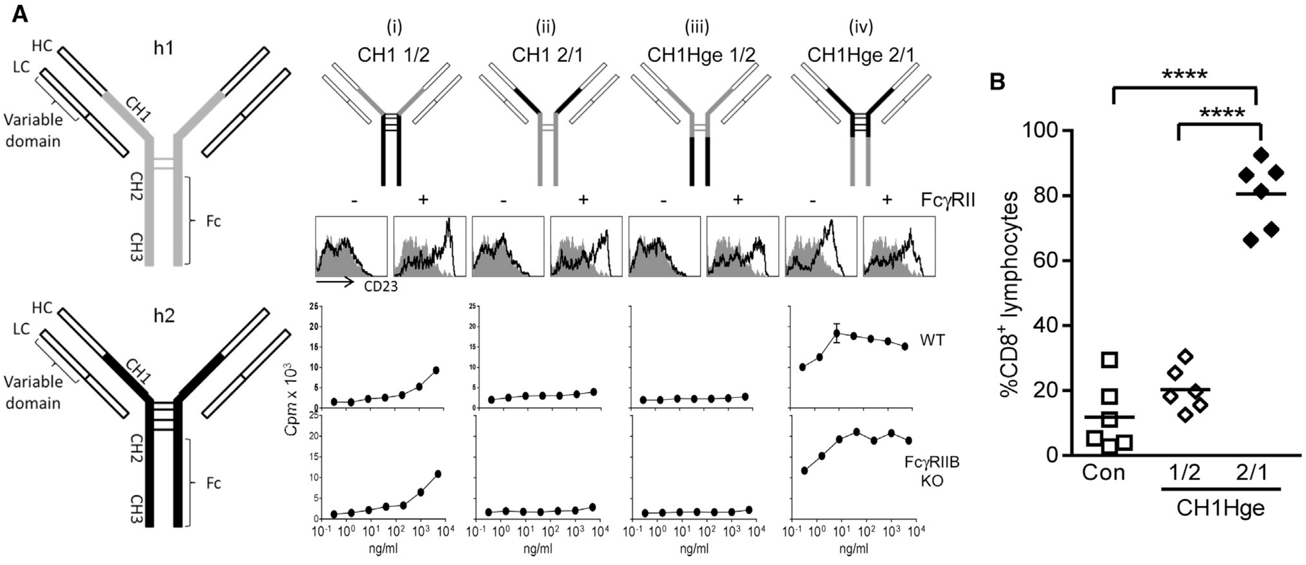

Figure 4. The CH1 and Hinge Regions Confer Activity to ChiLob7/4 h2

(A) Schematics of ChiLob 7/4 h1 and ChiLob 7/4 h2 (left) and mutants (top) where the CH1 (i, CH1 1/2 and ii, CH1 2/1) or CH1 and hinge regions (iii, CH1Hge 1/2 and iv, CH1Hge 2/1) of h1 and h2 were swapped. Middle: CD23 expression on human B cells in the absence or presence of FcγRIIB-expressing crosslinking cells. Bottom: hCD40 Tg FcγRIIB WT or KO B cell proliferation in response to the chimeric mAb (mean and range of duplicates).

(B) OTI responses in hCD40 Tg mice (n = 3) treated with the indicated mAb determined as in Figure 2E. Combined data from two experiments. ****$p < 0.0001$. See also Figure S3.

C127S mutant (Figure 6D, HC C127S) showed increased activity relative to native ChiLob 7/4 h2 at high concentrations for both FcγRIIB WT and KO cells (Figure 6C, iv). These combined data suggested that the FcγR-independent agonistic activity of h2 is contingent upon the precise conformation of disulfide bonds in its hinge and CH1 domains, and specifically on its ability to adopt the more compact h2B form.

Immune activation through CD40 ligation appears to require receptor clustering in the cell membrane to allow tumor necrosis factor receptor associated factor recruitment and propagation of downstream intracellular signals. Many of the effects are mediated through nuclear factor κB (NFκB) activation (Elgueta et al., 2009). Experiments with both primary hCD40 Tg B cells (Figure 6E) and transformed human Ramos B cells (Figure S4D) revealed a much greater capacity of ChiLob 7/4 h2B to activate NFκB as reflected by greatly enhanced inhibitor of NFκB (IκB) phosphorylation after cell stimulation compared to ChiLob 7/4 h2A. This is consistent with an ability of the more compact h2B to promote clustering of CD40 in the membrane, leading to NFκB signaling and cellular activation.

Mutagenesis Produces a Range of IgG2 Agonistic Activities

Further studies revealed ChiLob 7/4 h2 could be manipulated to achieve a range of agonistic activities. Mutation of LC Cys214 to Ser prevented the LC-HC disulfide linkage, resulting in two peaks on nrCE-SDS (Figure 6D, LC C214S). However, the mAb remained intact under nondenaturing conditions, with no reduction in binding to CD40 as measured by SPR (Figure 6F) or flow cytometry (Figure S4E). LC C214S caused an increase in hCD40 Tg B cell proliferation similar to that of the C127S mutant (Figure 6C, i). However, LC C214S combined with HC 232S gave a profile similar to that of native h2 where activity was greatly reduced at high concentrations (Figure 6C, ii), whereas LC C214S combined with HC C233S provided maximum activity similar to that of skewed h2B (Figure 6C, iii versus i).

As noted in the experiments described above, native ChiLob 7/4 h2 (a mixture of isoforms) produced a characteristic bell-shaped concentration curve when used to stimulate B cells in vitro, in which B cell responses were lower at high concentrations (e.g., Figures 2D, 3B, and 6C, i). This effect was largely lost with pure h2B, where high concentrations remained fully active (Figure 6C), but it was recapitulated with a 1:1 mixture of h2A and h2B (Figure 6G). Given the complete lack of response to the h2A form, this suggests h2A can block the activity of h2B, reducing its potency at high concentrations and suggesting that h2A possesses a certain level of antagonistic activity.

Finally, differences in ChiLob 7/4 h2A and ChiLob 7/4 h2B activity were recapitulated in vivo, where h2B caused significantly greater expansion of both OVA-specific CD8 T cells (Figure 6H) and OVA-specific IgG (Figure 6I) than h2A in hCD40 Tg FcγRIIB KO mice. Similar differences in in vivo activity were observed for h2A and h2B skewed forms of the CH1Hge 2/1 mutant (Figures S4F and S4G). In conclusion, our data demonstrate that manipulation of the disulfide structure of h2 may enable the production of therapeutic agents with defined and diverse immunostimulatory function that, importantly, is independent of the presence of FcγR in target tissues.

DISCUSSION

The recent clinical success of Ab immunotherapy has intensified the search for more effective immunostimulatory mAb in cancer treatment (Brahmer et al., 2012; Hodi et al., 2010; Topalian et al.,

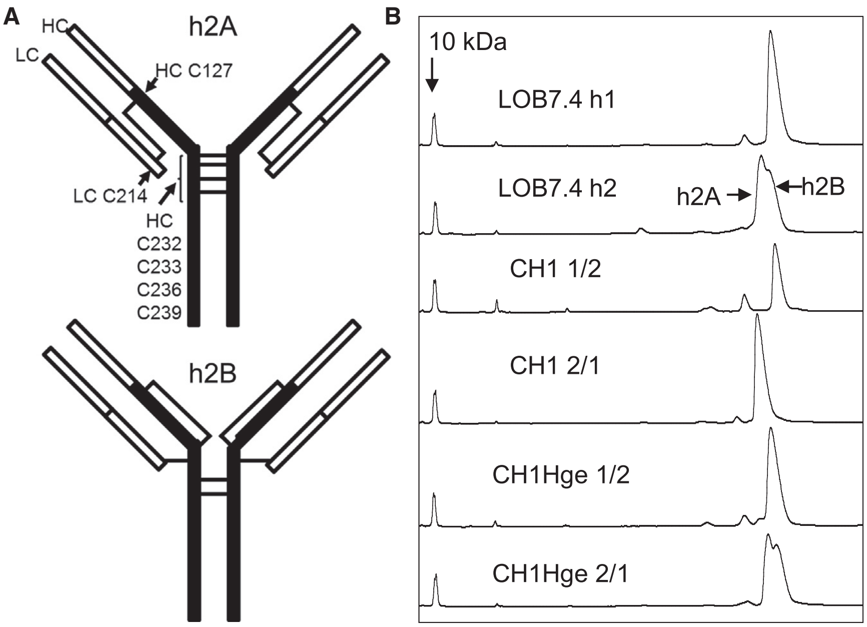

Figure 5. Disulfide Shuffling in the Human IgG2 Hinge

(A) Schematic representation of differently disulfide-linked h2 isoforms as described, for example, by Martinez et al. (2008).

(B) nrCE-SDS profiles of the indicated ChiLob 7/4 mAb. Positions of h2A and h2B and a 10 kDa standard are indicated.

2012; Wolchok et al., 2013). A large body of data now indicates that isotype selection is crucial as it dictates differential FcγR interactions that direct events after antigen binding (Nimmerjahn and Ravetch, 2012; White et al., 2013). Manipulation of the mAb Fc has already been used as a way to enhance selected interactions and increase therapeutic potency (Li and Ravetch, 2012, 2013; White et al., 2013). The data in this report show that isotype-dependent, FcγR-independent mechanisms may also be important determinants of activity.

The finding by us and others that agonistic anti-CD40, at least in murine models, requires binding to FcγRIIB (Li and Ravetch, 2011; White et al., 2011) poses a challenge when developing agents for human use as human IgG bind to FcγRIIB with very low affinity, particularly as monomers (Bruhns et al., 2009). Although Fc engineering can enhance FcγRIIB interaction and improve activity (Li and Ravetch, 2012, 2013; White et al., 2013), this approach is limited by the fact that FcγRIIB may not always be available for crosslinking within the tumor microenvironment and may also result in adverse events when FcγRIIB is engaged on endothelial cells (Xu et al., 2003). The demonstration in this report that mAb of the h2 isotype possess FcγR-independent agonistic activity is thus significant as it provides the opportunity to develop reagents that are agonistic regardless of target cell location.

The human IgG2 isotype is unique in its ability to rearrange disulfide bonds within its hinge and CH1 domains after synthesis, resulting in a range of isoforms with distinct conformations (Allen et al., 2009; Dillon et al., 2008; Martinez et al., 2008; Wypych et al., 2008). It is believed to be synthesized with a classical, flexible IgG structure (its "A" form) containing four inter-HC hinge disulfide bonds. Over time this is converted through a series of intermediates to a more compact "B" form in which the LC and HC CH1 are disulfide bonded to HC hinge cysteines 232 and 233 (Allen et al., 2009; Dillon et al., 2008; Liu et al., 2008; Martinez et al., 2008; Wypych et al., 2008). Using both a chemical skewing approach and a series of genetically engineered mAbs, we have demonstrated that the agonistic activity of ChiLob 7/4 h2 is dependent upon its ability to adopt the h2B form. Moreover, mutation of specific cysteines or combinations thereof could lock it into conformations with different degrees of agonistic activity.

Of note, the selected LC appears to affect the ability of h2 to adopt different conformations, with disulfide shuffling permitted by kappa but not lambda LCs (Dillon et al., 2008). Consistent with this, all mAbs used in our study contained the kappa LC. Of further interest, the existence of disulfide linked h2 dimers has been described in human blood (Yoo et al., 2003). However, as also reported by others using recombinant mAbs (Martinez et al., 2008), we found no evidence of dimers in any of our mAb preparations, as revealed by nrCE-SDS.

Previous attempts to investigate the functional impact of h2A and h2B isoforms have not revealed differences in FcγR or C1q engagement (Lightle et al., 2010), or consistent differences in Ag binding or the ability to block receptor-ligand interactions, where if anything h2B is less active (Dillon et al., 2008; Guo et al., 2008; Martinez et al., 2008). Similarly, we did not observe any difference in the avidity of ChiLob 7/4 h2A and h2B forms for CD40 when measured by SPR or by flow cytometry. Combined with the very similar affinities of ChiLob 7/4 h1 and ChiLob 7/4 h2 Fab′ for CD40, it seems highly unlikely that changes in affinity can explain the very different properties of h2A and h2B. Interestingly, Liu et al. (2008) also showed in patients the natural conversion from the A-to-B form over a number of days that did not result from a change in half-life. In the current study we assessed the agonistic activity of mAbs engaging immune coreceptors, where we know that receptor clustering is a mandatory requirement to initiate downstream immune activation (Elgueta et al., 2009). Finding an Ab format that can achieve such crosslinking without FcγR engagement is both unexpected and unexplained, as almost all agonistic mAbs described to date, including the anti-CD28 superagonist TGN1412, which caused catastrophic toxicity in healthy volunteers in 2006 (Suntharalingam et al., 2006), require FcγR crosslinking for activity (Bartholomaeus et al., 2014). We speculate that the agonistic properties of h2B result from its unusual compact conformation where the Fab′ arms are rotated down close to the Fc region of the Ab. This may allow close "packing" of adjacent receptors engaged in the plane of the membrane. The lack of flexibility in h2B may also hold receptors in a more rigid lattice that favors efficient downstream signaling. An extension of this may be that h2B, unlike h2A, can stabilize receptors in preexisting clusters (Smulski

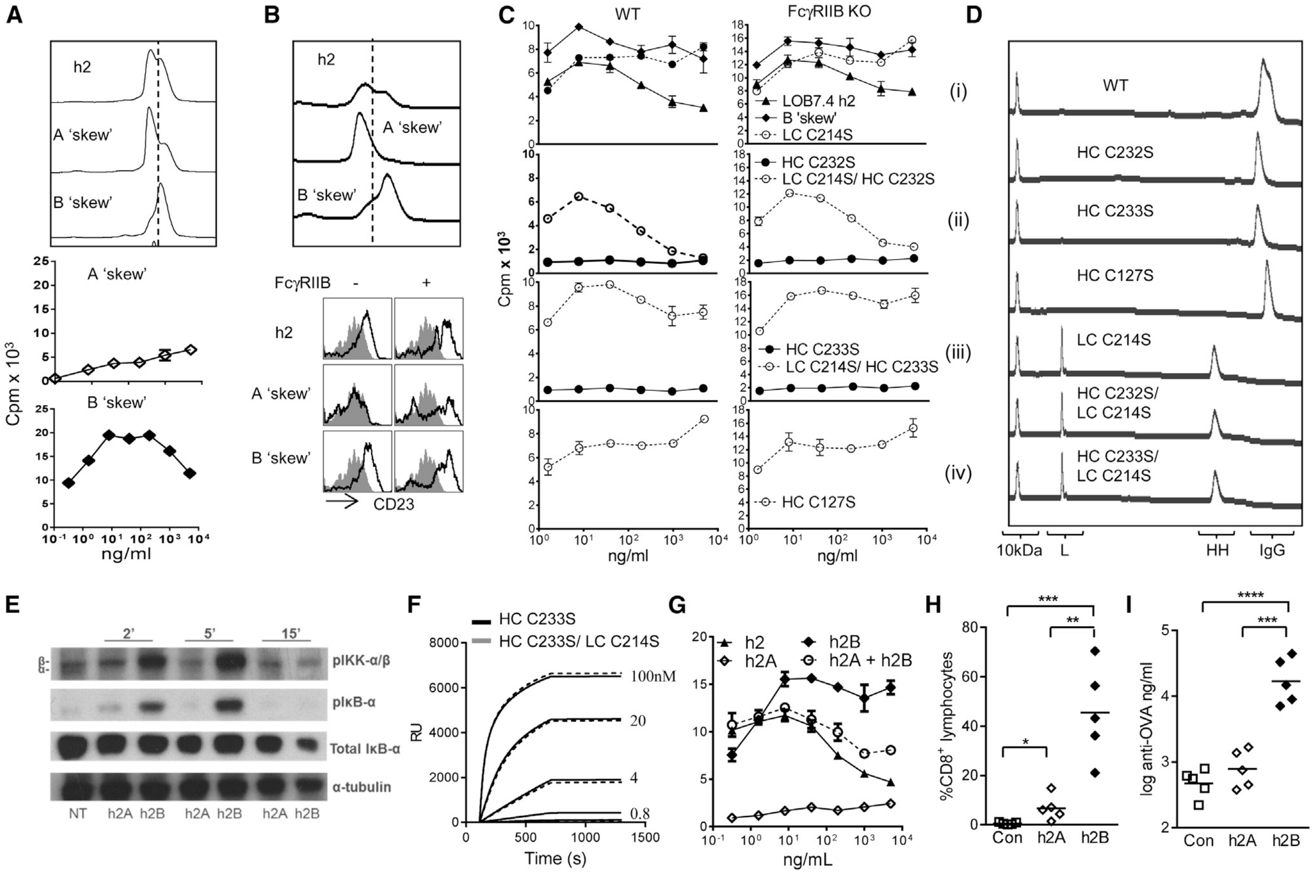

Figure 6. Mutagenesis Generates a Range of ChiLob 7/4 h2 Agonistic Forms

(A) nrCE-SDS profiles (top) and hCD40 Tg B cell proliferation in response to "skewed" h2A and h2B ChiLob 7/4 (mean and range of duplicates, one of three experiments).

(B) nrCE-SDS profiles (top) and mouse B cell activation assessed by CD23 upregulation in the presence and absence of FcγRIIB-expressing crosslinking cells (bottom) in response to skewed 3/23 h2 (one of three experiments shown).

(C) Proliferation of hCD40 Tg B cells that were WT or KO for FcγRIIB in response to ChiLob 7/4 mutants (mean and range of duplicates from one of at least three experiments).

(D) nrCE-SDS profiles of the indicated ChiLob 7/4 mutants. Positions of whole IgG, HC-HC complexes (HH), free LC (L), and 10 kDa marker are shown.

(E) Western blot of lysates from hCD40 Tg mouse B cells treated with ChiLob 7/4 h2A and ChiLob 7/4 h2B at 1 μg/ml for the indicated times and probed with Ab specific for phospho (p)-IκB kinase (IKK) α/β, pIκB-α, or IκB-α. Anti-tubulin was used as a loading control.

(F) SPR of ChiLob 7/4 h2 mutants (100, 20, 4, 0.8, and 0.16 nM) binding to hCD40 immobilized at 8,000 response units (RU).

(G) hCD40 Tg B cell proliferation with ChiLob 7/4 h2, h2A (HC C233S), skewed h2B, or a 1:1 mixture of h2A:h2B. Mean and range of duplicates from one of more than five experiments.

(H and I) OVA-specific OTI CD8 T cell responses (H) and day 18 serum Ab responses (I) in hCD40 Tg FcγRIIB KO mice (n = 5) immunized with OVA plus 100 μg of ChiLob 7/4 C233S (h2A) or skewed h2B. Results from one of two experiments. $^{**}p < 0.01$, $^{***}p < 0.001$.

See also Figure S4.

et al., 2013), while the flexible h2A may cause dissociation of these clusters. As h2 is the predominant isotype produced in response to bacterial polysaccharides (Barrett and Ayoub, 1986), the ability to form h2B may be an evolutionary response driven by the need to engage these repetitive, closely packed epitopes. The ability of h2B to engage such closely packed epitopes is the subject of our ongoing research.

The characteristic bell-shaped curves observed in this study when mixtures of anti-CD40 h2A and h2B were used to stimulate B cells whereby decreased activity was seen at higher concentrations (e.g., Figure 2D) may reflect the ability of the more flexible h2A form to outcompete crosslinking by the more structurally constrained h2B form. This could perhaps be due to the flexible h2A binding more efficiently to target molecules that are continually moving in a fluid plasma membrane. Ongoing studies aim to determine the precise configurations of the different forms of h2 to shed light on their precise modes of action. The observation that h2 constant regions also conferred FcγR-independent activity on another anti-hCD40 mAb, SGN40, as well as mAb directed against other receptors (4-1BB and CD28) suggests this may be a general property of this restricted conformation.

In vivo experiments in hCD40Tg mice clearly demonstrated the different mechanisms of action of ChiLob 7/4 when

administered as a chimeric m1 versus h2 mAb. In both cases immunostimulation was observed; however, the activity of m1 was dependent upon FcγRIIB expression, whereas that of h2 was completely independent of FcγR interaction. This raises the possibility of further enhancing activity by engineering reagents to simultaneously engage both mechanistic pathways; for example, a chimeric CH1Hge 2/1 containing the S267E/L328F mutation to increase FcγRIIB affinity (Chu et al., 2008). This is the subject of ongoing investigation. In addition, although h2 activity in our study was FcγR independent, it will be important to determine whether its activity can be influenced in vivo by the presence of human FcγR that may bind h2 immobilized on the cell surface with sufficient affinity to allow crosslinking, particularly in patients expressing FcγRIIA-131H or FcγRIIIA-158V (Lux et al., 2013).

Importantly, the most agonistic of the anti-CD40 mAbs in clinical trial to date is CP870,893, which is an h2, unlike the less agonistic ChiLob 7/4 and SGN40, which are both h1. The maximum tolerated dose of CP870,893 is at least 10-fold lower than that for ChiLob 7/4 or SGN40 (Vonderheide and Glennie 2013), and promising clinical data are emerging with this agent in both pancreatic cancer and metastatic melanoma patients (Bajor et al., 2014; Beatty et al., 2013). As in the current study, Richman and Vonderheide (2014) recently demonstrated that the in vitro agonistic activity of CP870,893 is both Fc independent and FcγR independent. This is significant as it suggests that FcγR-independent pathways can deliver results in a clinical setting, and our current findings might go some way toward explaining the unusual potency of CP870,893.

The data presented have profound implication for the development of agonistic mAb-based therapeutics. Equipped with these insights, it should be possible to manipulate the disulfide bond configuration of h2 to control the activity and toxicity of mAbs directed against a range of immune receptors, thereby permitting the fine-tuning of biological function and the subsequent development of novel therapeutics independent of FcγR interaction.

EXPERIMENTAL PROCEDURES

Mice

C57Bl/6 and RAG$^{-/-}$ mice were from Charles River Laboratories. Other genetically altered strains (all on C57BL/6 background) were FcγRIIB$^{-/-}$ (Boross et al., 2011), OTI TCR Tg (kindly provided by Dr. Matthias Merkenschlager, Imperial College), and human CD40 Tg (hCD40 Tg) (kindly provided by Randolph Noelle, Kings College) (Ahonen et al., 2002). Human CD40 Tg/FcγRIIB$^{-/-}$ and γ chain$^{-/-}$ mice were generated by crossbreeding with genotypes confirmed by flow cytometry. Animals were bred and housed in a local animal facility and were used at approximately 8–12 weeks of age. All experiments were reviewed and approved by both the Science Review Group and the Animal Welfare and Ethical Review Board, University of Southampton, and were carried out under UK Home Office license numbers PPL30/2451 and PPL30/2964.

Antibodies and Reagents

The following hybridomas were used: anti-human CD23 (MHM6) was from J. Gordon (University of Birmingham). Anti-human FcγRII (AT10) that binds both FcγRIIA and -IIB (Greenman et al., 1991), anti-human FcγRIIB (KB61), anti-human CD40 (ChiLob 7/4; Chowdhury et al., 2014), and anti-human 4-1BB were produced in house using conventional hybridoma technology. Anti-mouse CD23-phycoerythrin (PE) was from BD Biosciences. For OTI cell staining, APC-anti-mouse CD8α (clone 53-6.7; BD Biosciences) and PE-labeled SIINFEKL tetramers produced in house as described previously (White et al., 2011) were used. Flow cytometry was performed using an FACSCalibur system (BD Biosciences). Chicken OVA was from Sigma-Aldrich. Endotoxin-free OVA was from Profos.

Chimeric Antibodies

DNA constructs encoding HC and LC (kappa) variable regions of various mAbs were either amplified from hybridoma by PCR reactions or synthesized by Genewiz. The anti-CD40 mAb SGN40-Soton and the anti-CD28 TGN-Soton were produced using published sequences (Drachman et al., 2007 and Hanke et al., 2009, respectively). Details of mAb purification and quality control methods can be found in Supplemental Experimental Procedures.

Immunization and Assessment of Immune Responses

Mice were immunized as detailed for individual experiments via tail vein injection in 200 μl of PBS. Serum anti-OVA Ab levels were determined by ELISA (White et al., 2010). In some experiments, 3×10^5 splenic OVA-specific CD8 (OTI) T cells were given via tail vein the day before immunization.

Tumor Therapy

For vaccination against the OVA-expressing thymoma EG7, mice were adoptively transferred with 5×10^4 OTI cells on day −6 and then received 0.5 mg of Sigma OVA + 100 μg of anti-CD40 mAb on day −5. The mice were challenged with 5×10^5 EG7 tumor cells subcutaneously on day 0. Tumor growth was monitored, and mice were sacrificed when the humane endpoint was reached (15 mm mean tumor diameter when taking the two greatest perpendicular measurements). B cell lymphoma (BCL_1) therapies were performed as described previously (White et al., 2014). In brief, mice were inoculated via tail vein with 1×10^4 BCL_1 cells on day 0. On day 14 (when the tumor was well established and represented 5%–10% spleen weight), mice were treated with a single 100 μg dose of anti-CD40.

Cell Activation and Proliferation

Details of the isolation and assays to assess the activation and/or proliferation of DCs, B cells, and T cells can be found in Supplemental Experimental Procedures. For isolation of human Langerhans cells, skin specimens were acquired from healthy individuals. For peripheral blood B cells, anonymized leukocyte reduction system cones from healthy adult subjects were obtained from the National Blood Transfusion Service, Southampton, UK. All human samples were collected following informed consent and used under ethical approval (National Research Ethics Service, UK) in accordance with the Declaration of Helsinki.

SPR

A Biacore T100 system (GE Healthcare) was used to assay the interaction between soluble Fcγ receptors and ChiLob 7/4 mAb isotypes, as well as between soluble mAb and immobilized CD40, as described in Supplemental Experimental Procedures.

Statistical Analyses

Student's t tests (unpaired, two-tailed) and survival analyses (log rank Mantel-Cox tests) were performed using Prism software (GraphPad). For comparison of Ab responses, data were log transformed before analysis. In some cases data from multiple experiments were combined. However, this was not always possible as although relative differences remained the same between experiments, absolute measures often varied too much to allow combination. When this was the case, a single representative experiment is shown, with the number of experiments performed stated in the figure legend.

SUPPLEMENTAL INFORMATION

Supplemental Information includes Supplemental Experimental Procedures and five figures and can be found with this article online at http://dx.doi.org/10.1016/j.ccell.2014.11.001.

ACKNOWLEDGMENTS

We thank Marion Brown, Neil Barclay, Geoffrey Hale, and Ivo Tews for helpful discussions and Randolph Noelle for the hCD40 Tg mice. This research was

supported by Cancer Research UK and an EU Framework HEALTH-2013-INNOVATION grant. M.S.C. acts as a consultant for BioInvent and has received research funding from BioInvent and Roche.

Received: August 6, 2014
Revised: October 3, 2014
Accepted: November 4, 2014
Published: December 11, 2014

REFERENCES

Advani, R., Forero-Torres, A., Furman, R.R., Rosenblatt, J.D., Younes, A., Ren, H., Harrop, K., Whiting, N., and Drachman, J.G. (2009). Phase I study of the humanized anti-CD40 monoclonal antibody dacetuzumab in refractory or recurrent non-Hodgkin's lymphoma. J. Clin. Oncol. *27*, 4371–4377.

Ahonen, C., Manning, E., Erickson, L.D., O'Connor, B., Lind, E.F., Pullen, S.S., Kehry, M.R., and Noelle, R.J. (2002). The CD40-TRAF6 axis controls affinity maturation and the generation of long-lived plasma cells. Nat. Immunol. *3*, 451–456.

Allen, M.J., Guo, A., Martinez, T., Han, M., Flynn, G.C., Wypych, J., Liu, Y.D., Shen, W.D., Dillon, T.M., Vezina, C., and Balland, A. (2009). Interchain disulfide bonding in human IgG2 antibodies probed by site-directed mutagenesis. Biochemistry *48*, 3755–3766.

Angal, S., King, D.J., Bodmer, M.W., Turner, A., Lawson, A.D., Roberts, G., Pedley, B., and Adair, J.R. (1993). A single amino acid substitution abolishes the heterogeneity of chimeric mouse/human (IgG4) antibody. Mol. Immunol. *30*, 105–108.

Bajor, D.L., Xu, X., Torigian, D.A., Mick, R., Garcia, L.R., Richman, L.P., Desmarais, C., Nathanson, K.L., Schuchter, L.M., Kalos, M., and Vonderheide, R.H. (2014). Immune activation and a 9-year ongoing complete remission following CD40 antibody therapy and metastasectomy in a patient with metastatic melanoma. Cancer Immunol. Res. *2*, 1051–1058.

Barrett, D.J., and Ayoub, E.M. (1986). IgG2 subclass restriction of antibody to pneumococcal polysaccharides. Clin. Exp. Immunol. *63*, 127–134.

Bartholomaeus, P., Semmler, L.Y., Bukur, T., Boisguerin, V., Römer, P.S., Tabares, P., Chuvpilo, S., Tyrsin, D.Y., Matskevich, A., Hengel, H., et al. (2014). Cell contact-dependent priming and Fc interaction with CD32+ immune cells contribute to the TGN1412-triggered cytokine response. J. Immunol. *192*, 2091–2098.

Beatty, G.L., Chiorean, E.G., Fishman, M.P., Saboury, B., Teitelbaum, U.R., Sun, W., Huhn, R.D., Song, W., Li, D., Sharp, L.L., et al. (2011). CD40 agonists alter tumor stroma and show efficacy against pancreatic carcinoma in mice and humans. Science *331*, 1612–1616.

Beatty, G.L., Torigian, D.A., Chiorean, E.G., Saboury, B., Brothers, A., Alavi, A., Troxel, A.B., Sun, W., Teitelbaum, U.R., Vonderheide, R.H., and O'Dwyer, P.J. (2013). A phase I study of an agonist CD40 monoclonal antibody (CP-870,893) in combination with gemcitabine in patients with advanced pancreatic ductal adenocarcinoma. Clin. Cancer Res. *19*, 6286–6295.

Boross, P., Arandhara, V.L., Martin-Ramirez, J., Santiago-Raber, M.L., Carlucci, F., Flierman, R., van der Kaa, J., Breukel, C., Claassens, J.W., Camps, M., et al. (2011). The inhibiting Fc receptor for IgG, FcγRIIB, is a modifier of autoimmune susceptibility. J. Immunol. *187*, 1304–1313.

Brahmer, J.R., Tykodi, S.S., Chow, L.Q., Hwu, W.J., Topalian, S.L., Hwu, P., Drake, C.G., Camacho, L.H., Kauh, J., Odunsi, K., et al. (2012). Safety and activity of anti-PD-L1 antibody in patients with advanced cancer. N. Engl. J. Med. *366*, 2455–2465.

Bruhns, P., Iannascoli, B., England, P., Mancardi, D.A., Fernandez, N., Jorieux, S., and Daëron, M. (2009). Specificity and affinity of human Fcgamma receptors and their polymorphic variants for human IgG subclasses. Blood *113*, 3716–3725.

Bulliard, Y., Jolicoeur, R., Windman, M., Rue, S.M., Ettenberg, S., Knee, D.A., Wilson, N.S., Dranoff, G., and Brogdon, J.L. (2013). Activating Fc γ receptors contribute to the antitumor activities of immunoregulatory receptor-targeting antibodies. J. Exp. Med. *210*, 1685–1693.

Bulliard, Y., Jolicoeur, R., Zhang, J., Dranoff, G., Wilson, N.S., and Brogdon, J.L. (2014). OX40 engagement depletes intratumoral Tregs via activating FcγRs, leading to antitumor efficacy. Immunol. Cell Biol. *92*, 475–480.

Chowdhury, F., Johnson, P.W., Glennie, M.J., and Williams, A.P. (2014). Ex vivo assays of dendritic cell activation and cytokine profiles as predictors of in vivo effects in an anti-human CD40 monoclonal antibody ChiLob 7/4 phase I trial. Cancer Immunol. Res. *2*, 229–240.

Chu, S.Y., Vostiar, I., Karki, S., Moore, G.L., Lazar, G.A., Pong, E., Joyce, P.F., Szymkowski, D.E., and Desjarlais, J.R. (2008). Inhibition of B cell receptor-mediated activation of primary human B cells by coengagement of CD19 and FcgammaRIIb with Fc-engineered antibodies. Mol. Immunol. *45*, 3926–3933.

Clynes, R.A., Towers, T.L., Presta, L.G., and Ravetch, J.V. (2000). Inhibitory Fc receptors modulate in vivo cytotoxicity against tumor targets. Nat. Med. *6*, 443–446.

Drachman, J., Law, C.-L., and Lewis, T. May 2007. Methods of using CD40 binding agents. U.S. patent WO/2007/075326.

Dillon, T.M., Ricci, M.S., Vezina, C., Flynn, G.C., Liu, Y.D., Rehder, D.S., Plant, M., Henkle, B., Li, Y., Deechongkit, S., et al. (2008). Structural and functional characterization of disulfide isoforms of the human IgG2 subclass. J. Biol. Chem. *283*, 16206–16215.

Elgueta, R., Benson, M.J., de Vries, V.C., Wasiuk, A., Guo, Y., and Noelle, R.J. (2009). Molecular mechanism and function of CD40/CD40L engagement in the immune system. Immunol. Rev. *229*, 152–172.

French, R.R., Chan, H.T., Tutt, A.L., and Glennie, M.J. (1999). CD40 antibody evokes a cytotoxic T-cell response that eradicates lymphoma and bypasses T-cell help. Nat. Med. *5*, 548–553.

French, R.R., Taraban, V.Y., Crowther, G.R., Rowley, T.F., Gray, J.C., Johnson, P.W., Tutt, A.L., Al-Shamkhani, A., and Glennie, M.J. (2007). Eradication of lymphoma by CD8 T cells following anti-CD40 monoclonal antibody therapy is critically dependent on CD27 costimulation. Blood *109*, 4810–4815.

Greenman, J., Tutt, A.L., George, A.J., Pulford, K.A., Stevenson, G.T., and Glennie, M.J. (1991). Characterization of a new monoclonal anti-Fc gamma RII antibody, AT10, and its incorporation into a bispecific F(ab')2 derivative for recruitment of cytotoxic effectors. Mol. Immunol. *28*, 1243–1254.

Guo, A., Han, M., Martinez, T., Ketchem, R.R., Novick, S., Jochheim, C., and Balland, A. (2008). Electrophoretic evidence for the presence of structural isoforms specific for the IgG2 isotype. Electrophoresis *29*, 2550–2556.

Hamaguchi, Y., Xiu, Y., Komura, K., Nimmerjahn, F., and Tedder, T.F. (2006). Antibody isotype-specific engagement of Fcgamma receptors regulates B lymphocyte depletion during CD20 immunotherapy. J. Exp. Med. *203*, 743–753.

Hanke, T., Trischler, M., and Guntermann, C. September 2009. Encoding a binding molecule specifically binding to a human CD28 molecule; comprising a nucleic acid sequence encoding a VH region and a nucleic acid sequence encoding a VL region with CDRs in a human immunoglobulin framework. U.S. patent US7585960.

Hanks, B.A., Jiang, J., Singh, R.A., Song, W., Barry, M., Huls, M.H., Slawin, K.M., and Spencer, D.M. (2005). Re-engineered CD40 receptor enables potent pharmacological activation of dendritic-cell cancer vaccines in vivo. Nat. Med. *11*, 130–137.

Hodi, F.S., O'Day, S.J., McDermott, D.F., Weber, R.W., Sosman, J.A., Haanen, J.B., Gonzalez, R., Robert, C., Schadendorf, D., Hassel, J.C., et al. (2010). Improved survival with ipilimumab in patients with metastatic melanoma. N. Engl. J. Med. *363*, 711–723.

Kohrt, H.E., Houot, R., Goldstein, M.J., Weiskopf, K., Alizadeh, A.A., Brody, J., Müller, A., Pachynski, R., Czerwinski, D., Coutre, S., et al. (2011). CD137 stimulation enhances the antilymphoma activity of anti-CD20 antibodies. Blood *117*, 2423–2432.

Kohrt, H.E., Houot, R., Weiskopf, K., Goldstein, M.J., Scheeren, F., Czerwinski, D., Colevas, A.D., Weng, W.K., Clarke, M.F., Carlson, R.W., et al. (2012). Stimulation of natural killer cells with a CD137-specific antibody enhances

trastuzumab efficacy in xenotransplant models of breast cancer. J. Clin. Invest. *122*, 1066–1075.

Kohrt, H.E., Colevas, A.D., Houot, R., Weiskopf, K., Goldstein, M.J., Lund, P., Mueller, A., Sagiv-Barfi, I., Marabelle, A., Lira, R., et al. (2014). Targeting CD137 enhances the efficacy of cetuximab. J. Clin. Invest. *124*, 2668–2682.

Kurai, J., Chikumi, H., Hashimoto, K., Yamaguchi, K., Yamasaki, A., Sako, T., Touge, H., Makino, H., Takata, M., Miyata, M., et al. (2007). Antibody-dependent cellular cytotoxicity mediated by cetuximab against lung cancer cell lines. Clin. Cancer Res. *13*, 1552–1561.

Li, F., and Ravetch, J.V. (2011). Inhibitory Fcγ receptor engagement drives adjuvant and anti-tumor activities of agonistic CD40 antibodies. Science *333*, 1030–1034.

Li, F., and Ravetch, J.V. (2012). Apoptotic and antitumor activity of death receptor antibodies require inhibitory Fcγ receptor engagement. Proc. Natl. Acad. Sci. USA *109*, 10966–10971.

Li, F., and Ravetch, J.V. (2013). Antitumor activities of agonistic anti-TNFR antibodies require differential FcγRIIB coengagement in vivo. Proc. Natl. Acad. Sci. USA *110*, 19501–19506.

Lightle, S., Aykent, S., Lacher, N., Mitaksov, V., Wells, K., Zobel, J., and Oliphant, T. (2010). Mutations within a human IgG2 antibody form distinct and homogeneous disulfide isomers but do not affect Fc gamma receptor or C1q binding. Protein Sci. *19*, 753–762.

Liu, Y.D., Chen, X., Enk, J.Z., Plant, M., Dillon, T.M., and Flynn, G.C. (2008). Human IgG2 antibody disulfide rearrangement in vivo. J. Biol. Chem. *283*, 29266–29272.

Lux, A., Yu, X., Scanlan, C.N., and Nimmerjahn, F. (2013). Impact of immune complex size and glycosylation on IgG binding to human FcγRs. J. Immunol. *190*, 4315–4323.

Martinez, T., Guo, A., Allen, M.J., Han, M., Pace, D., Jones, J., Gillespie, R., Ketchem, R.R., Zhang, Y., and Balland, A. (2008). Disulfide connectivity of human immunoglobulin G2 structural isoforms. Biochemistry *47*, 7496–7508.

Moran, A.E., Kovacsovics-Bankowski, M., and Weinberg, A.D. (2013). The TNFRs OX40, 4-1BB, and CD40 as targets for cancer immunotherapy. Curr. Opin. Immunol. *25*, 230–237.

Nimmerjahn, F., and Ravetch, J.V. (2005). Divergent immunoglobulin g subclass activity through selective Fc receptor binding. Science *310*, 1510–1512.

Nimmerjahn, F., and Ravetch, J.V. (2012). Translating basic mechanisms of IgG effector activity into next generation cancer therapies. Cancer Immun. *12*, 13–19.

Richman, L.P., and Vonderheide, R.H. (2014). Role of crosslinking for agonistic CD40 monoclonal antibodies as immune therapy of cancer. Cancer Immunol. Res. *2*, 19–26.

Ryazantsev, S., Tischenko, V., Nguyen, C., Abramov, V., and Zav'yalov, V. (2013). Three-dimensional structure of the human myeloma IgG2. PLoS ONE *8*, e64076.

Sanchez, P.J., McWilliams, J.A., Haluszczak, C., Yagita, H., and Kedl, R.M. (2007). Combined TLR/CD40 stimulation mediates potent cellular immunity by regulating dendritic cell expression of CD70 in vivo. J. Immunol. *178*, 1564–1572.

Simpson, T.R., Li, F., Montalvo-Ortiz, W., Sepulveda, M.A., Bergerhoff, K., Arce, F., Roddie, C., Henry, J.Y., Yagita, H., Wolchok, J.D., et al. (2013). Fc-dependent depletion of tumor-infiltrating regulatory T cells co-defines the efficacy of anti-CTLA-4 therapy against melanoma. J. Exp. Med. *210*, 1695–1710.

Sliwkowski, M.X., and Mellman, I. (2013). Antibody therapeutics in cancer. Science *341*, 1192–1198.

Smulski, C.R., Beyrath, J., Decossas, M., Chekkat, N., Wolff, P., Estieu-Gionnet, K., Guichard, G., Speiser, D., Schneider, P., and Fournel, S. (2013). Cysteine-rich domain 1 of CD40 mediates receptor self-assembly. J. Biol. Chem. *288*, 10914–10922.

Suntharalingam, G., Perry, M.R., Ward, S., Brett, S.J., Castello-Cortes, A., Brunner, M.D., and Panoskaltsis, N. (2006). Cytokine storm in a phase 1 trial of the anti-CD28 monoclonal antibody TGN1412. N. Engl. J. Med. *355*, 1018–1028.

Topalian, S.L., Hodi, F.S., Brahmer, J.R., Gettinger, S.N., Smith, D.C., McDermott, D.F., Powderly, J.D., Carvajal, R.D., Sosman, J.A., Atkins, M.B., et al. (2012). Safety, activity, and immune correlates of anti-PD-1 antibody in cancer. N. Engl. J. Med. *366*, 2443–2454.

Uchida, J., Hamaguchi, Y., Oliver, J.A., Ravetch, J.V., Poe, J.C., Haas, K.M., and Tedder, T.F. (2004). The innate mononuclear phagocyte network depletes B lymphocytes through Fc receptor-dependent mechanisms during anti-CD20 antibody immunotherapy. J. Exp. Med. *199*, 1659–1669.

Vonderheide, R.H., and Glennie, M.J. (2013). Agonistic CD40 antibodies and cancer therapy. Clin. Cancer Res. *19*, 1035–1043.

White, A.L., Tutt, A.L., James, S., Wilkinson, K.A., Castro, F.V., Dixon, S.V., Hitchcock, J., Khan, M., Al-Shamkhani, A., Cunningham, A.F., and Glennie, M.J. (2010). Ligation of CD11c during vaccination promotes germinal centre induction and robust humoral responses without adjuvant. Immunology *131*, 141–151.

White, A.L., Chan, H.T., Roghanian, A., French, R.R., Mockridge, C.I., Tutt, A.L., Dixon, S.V., Ajona, D., Verbeek, J.S., Al-Shamkhani, A., et al. (2011). Interaction with FcγRIIB is critical for the agonistic activity of anti-CD40 monoclonal antibody. J. Immunol. *187*, 1754–1763.

White, A.L., Chan, H.T., French, R.R., Beers, S.A., Cragg, M.S., Johnson, P.W., and Glennie, M.J. (2013). FcγRIIB controls the potency of agonistic anti-TNFR mAbs. Cancer Immunol. Immunother. *62*, 941–948.

White, A.L., Dou, L., Chan, H.T.C., Field, V.L., Mockridge, C.I., Moss, K., Williams, E., Butts, C., Al-Shamkhani, A., Cragg, M.S., et al. (2014). FcR dependence of agonistic CD40 antibody is related to anatomical location and can be overcome by antibody multimerisation. J. Immunol. *193*, 1828–1835.

Wilson, N.S., Yang, B., Yang, A., Loeser, S., Marsters, S., Lawrence, D., Li, Y., Pitti, R., Totpal, K., Yee, S., et al. (2011). An Fcγ receptor-dependent mechanism drives antibody-mediated target-receptor signaling in cancer cells. Cancer Cell *19*, 101–113.

Wolchok, J.D., Kluger, H., Callahan, M.K., Postow, M.A., Rizvi, N.A., Lesokhin, A.M., Segal, N.H., Ariyan, C.E., Gordon, R.A., Reed, K., et al. (2013). Nivolumab plus ipilimumab in advanced melanoma. N. Engl. J. Med. *369*, 122–133.

Wypych, J., Li, M., Guo, A., Zhang, Z., Martinez, T., Allen, M.J., Fodor, S., Kelner, D.N., Flynn, G.C., Liu, Y.D., et al. (2008). Human IgG2 antibodies display disulfide-mediated structural isoforms. J. Biol. Chem. *283*, 16194–16205.

Xu, Y., Szalai, A.J., Zhou, T., Zinn, K.R., Chaudhuri, T.R., Li, X., Koopman, W.J., and Kimberly, R.P. (2003). Fc gamma Rs modulate cytotoxicity of anti-Fas antibodies: implications for agonistic antibody-based therapeutics. J. Immunol. *171*, 562–568.

Yoo, E.M., Wims, L.A., Chan, L.A., and Morrison, S.L. (2003). Human IgG2 can form covalent dimers. J. Immunol. *170*, 3134–3138.

Zhang, B., Harder, A.G., Connelly, H.M., Maheu, L.L., and Cockrill, S.L. (2010). Determination of Fab-hinge disulfide connectivity in structural isoforms of a recombinant human immunoglobulin G2 antibody. Anal. Chem. *82*, 1090–1099.

Cancer Cell, Volume *27*

Supplemental Information

Conformation of the Human Immunoglobulin G2 Hinge Imparts Superagonistic Properties to Immunostimulatory Anticancer Antibodies

Ann L. White, H.T. Claude Chan, Ruth R. French, Jane Willoughby, C. Ian Mockridge, Ali Roghanian, Christine A. Penfold, Steven G. Booth, Ali Dodhy, Marta E. Polak, Elizabeth A. Potter, Michael R. Ardern-Jones, J. Sjef Verbeek, Peter W.M. Johnson, Aymen Al-Shamkhani, Mark S. Cragg, Stephen A. Beers, and Martin J. Glennie

SUPPLEMENTAL DATA

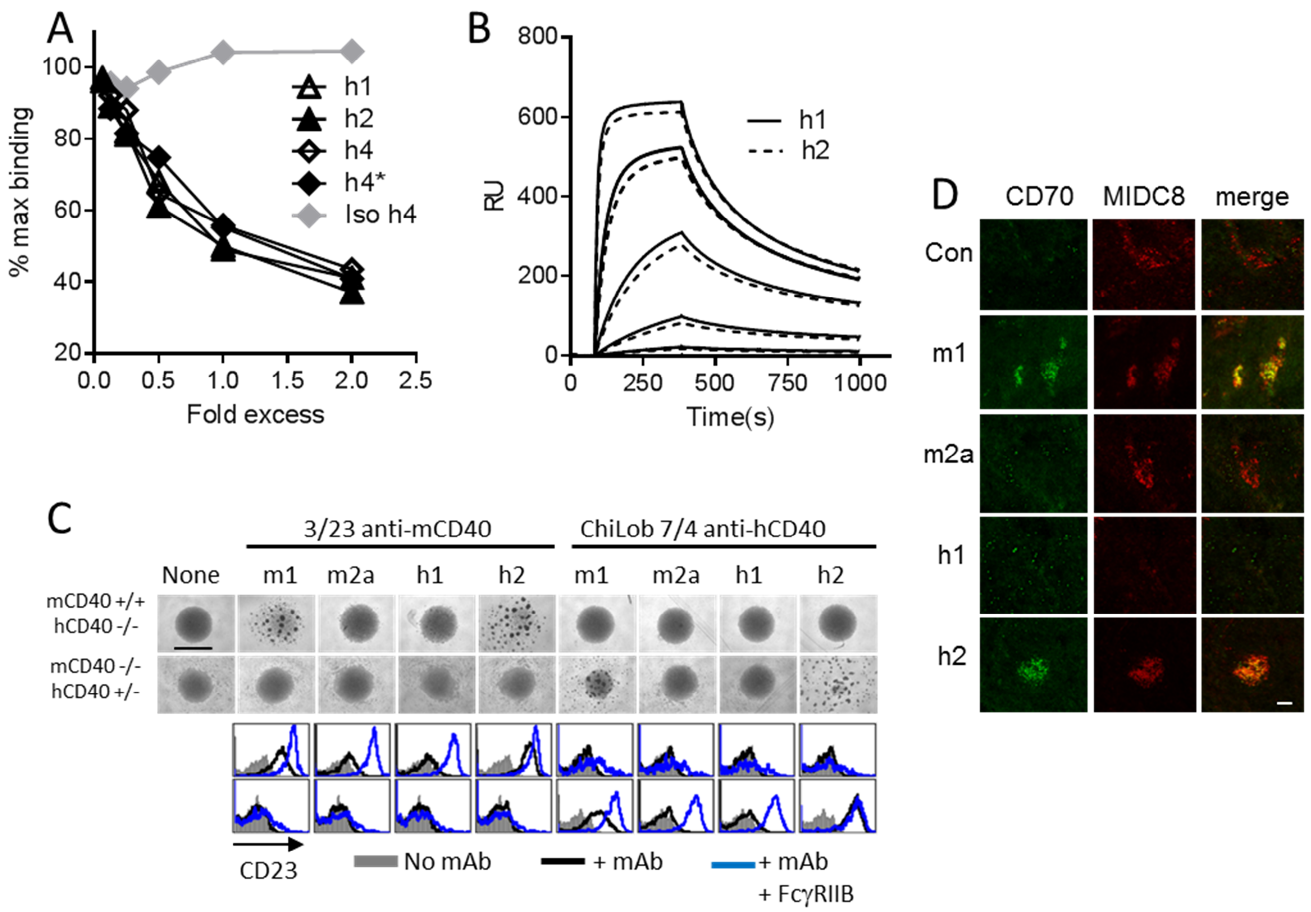

Figure S1. Related to Figure 1. Control experiments for effect of isotype on anti-CD40 activity.

(A) Purified hCD40Tg mouse B cells were incubated with 10 µg/ml ChiLob 7/4 h1-FITC pre-mixed with different concentrations of competing unlabelled ChiLob 7/4 mAb of different human isotypes. Flow cytometry was used to determine the level of ChiLob 7/4-FITC binding and is expressed as the % maximum MFI (no competing mAb present). A non-targeted human IgG4 isotype mAb (grey diamonds) was included as a non-competing control. **(B)** hCD40 was immobilised at 1000 RU and ChiLob 7/4 h1 (solid line) or h2 (broken line) Fab' fragments flowed over the chip at 640, 128, 25.6 and 1.024 nM. Affinities were calculated by fitting a 1:1 binding model and were 10.0 and 10.2 nM for h1 and h2, respectively. **(C)** Purified WT (mCD40+/+) or hCD40Tg/mCD40 KO (mCD40-/-, hCD40+/-) mouse B cells were incubated for 16 hr with anti-

mCD40 (3/23) or anti-hCD40 (ChiLob 7/4) of the indicated isotypes at 1 μg/ml. B-cell activation was assessed by homotypic adhesion (top; bar = 1mm) and CD23 upregulation (bottom; filled grey histograms, untreated cells; black line, treated with mAb alone; blue line, incubated with mAb + FcγRIIB over-expressing cross-linking cells). **(D)** Splenic sections from mice administered 100 μg of the indicated 3/23 isotypes stained for CD70 (green, left) and the DC marker MIDC8 (red, middle; merge on right). Bar = 100 μm. Results from 1 of 2 experiments shown.

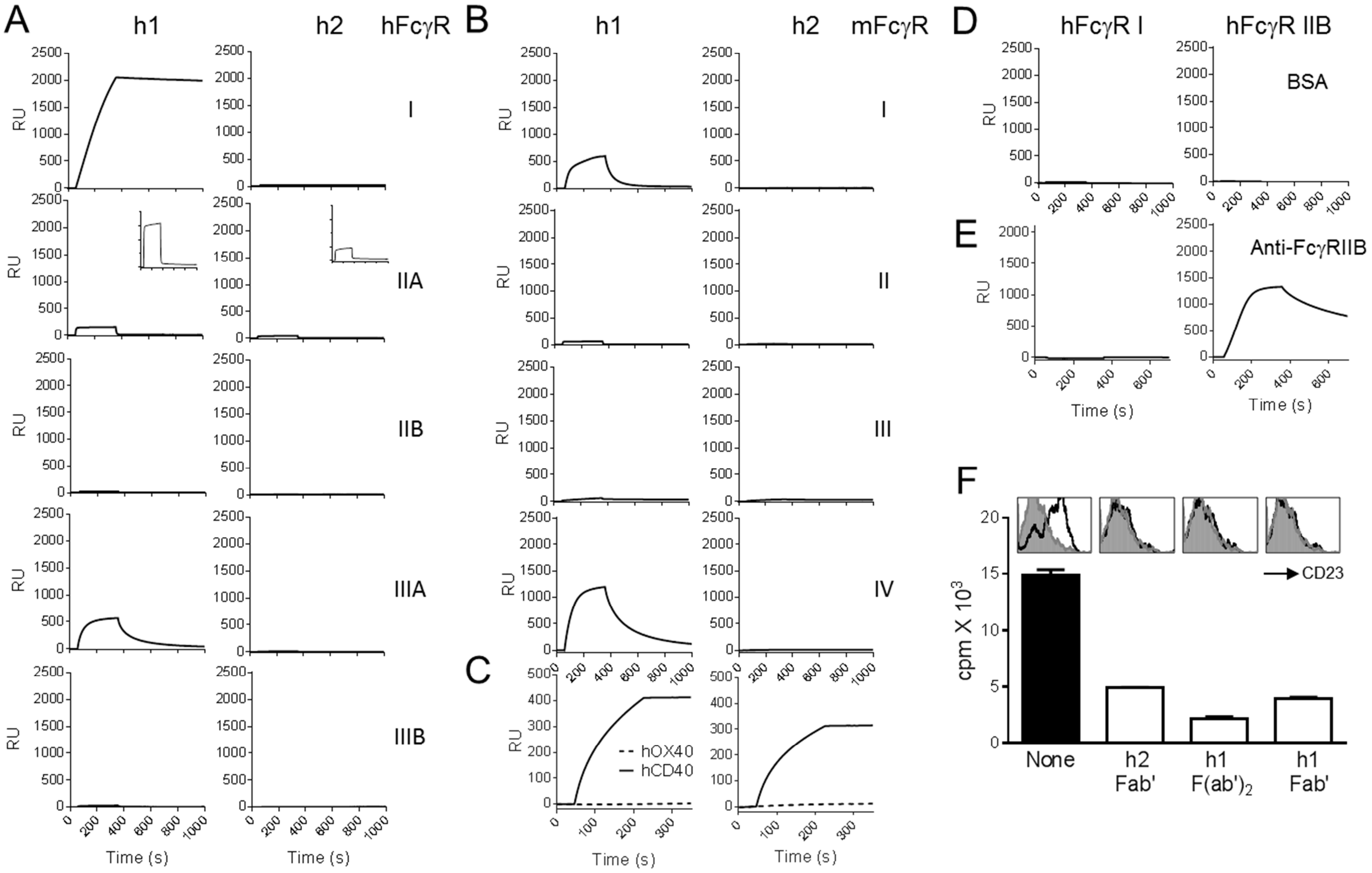

Figure S2. Related to Figure 2. ChiLob 7/4 h2 agonistic activity is FcγR independent. (A and B) SPR profiles to show binding of the indicated human (A) and mouse (B) FcγR at 100 nM to ChiLob 7/4 h1 or h2 immobilised at 15,000 RU. All profiles are presented on the same scale (Y axis to 2500 RU) to allow comparison of relative binding. The insets for FcγRIIA show the same data plotted with a Y axis scale of 200 RU to reveal low level binding. (C) To demonstrate integrity of the bound mAb, the binding of 100 nM hCD40 protein (solid line) or hOX40 protein (broken line) to the h1 and h2 mAb immobilised on the flow cells used in A and B was determined. (D) Background binding of hFcγRI and IIB to immobilised BSA. Similar results were obtained for all FcγR. This background was subtracted from the profiles in A and B. (E) To demonstrate integrity of the hFcγRIIB, anti-FcγRIIB-specific mAb (KB61) was immobilised and the binding of hFcγRI and IIB compared. (F) Purified hCD40Tg mouse B cells were incubated for 16 hours with 1 μg/ml ChiLob 7/4 h2 IgG alone (left, black bar) or in the presence of a 50-fold

excess of the indicated ChiLob 7/4 IgG fragments. CD23 expression was analysed by flow cytometry after 20 hr (top panel; grey histogram untreated cells, black line mAb treated cells) and B cell proliferation by ^{3}H thymidine incorporation after 5 days (bottom panel, mean and range of duplicate samples). Results from 1 of 2 experiments shown.

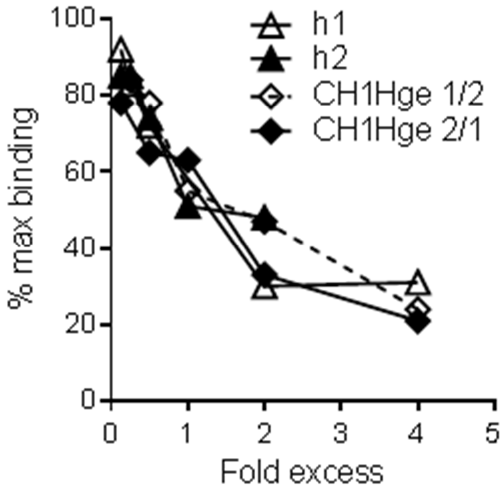

Figure S3. Related to Figure 4. ChiLob 7/4 switch mutants bind similarly to CD40.

Purified hCD40Tg mouse B cells were incubated with ChiLob 7/4 h1-FITC pre-mixed with different ratios of the indicated unlabelled mutants and analysed as in Figure S1A.

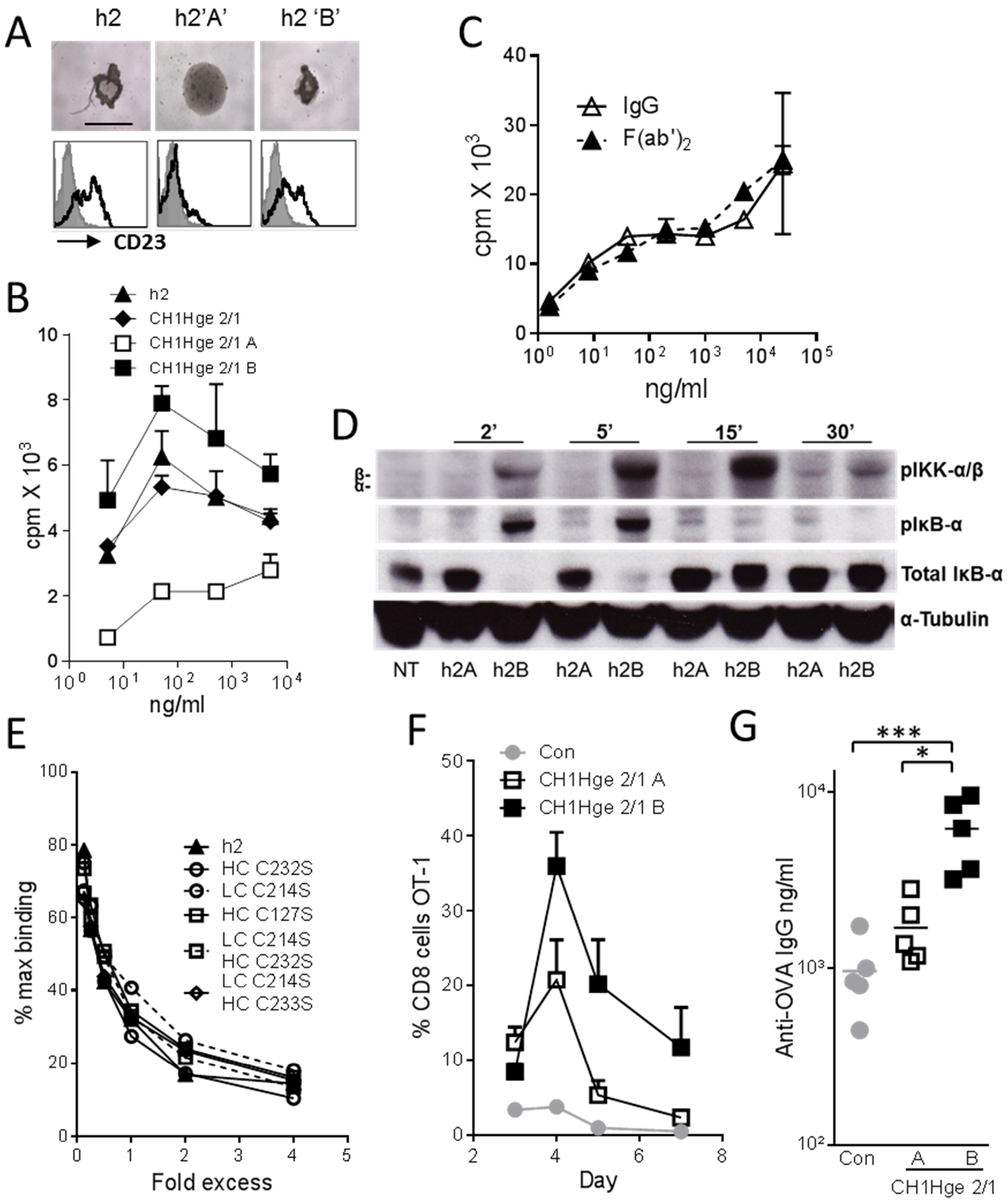

Figure S4. Related to Figure 6. Differential activity of ChiLob 7/4 h2A and h2B forms. (A) Activation of purified human B cells assessed by homotypic adhesion (top; bar = 1mm) and CD23 upregulation (bottom) after incubation with native, 'A' or 'B' skewed forms of ChiLob 7/4 h2 at 200 ng/ml for 16 hr. (B) Purified hCD40Tg B cells were incubated with the indicated concentrations of ChiLob 7/4 h2, CH1Hge 2/1 mutant or skewed forms of the mutant and proliferation measured as in Figure 6A (mean and range of duplicate samples). (C) hCD40Tg

mouse B cell proliferation in response to increasing concentrations of h2B skewed ChiLob 7/4 h2 IgG or $F(ab')_{2,}$ measured as in B. (D) Western blot of lysates from Ramos cells treated with ChiLob 7/4 h2A and h2B at 1 μg/ml for the indicated times and probed with Ab specific for phosph-IKKα/β, phospho IκB–α or IκB–α. Anti-tubulin was used as a loading control. (E) Purified hCD40Tg mouse B cells were incubated with ChiLob 7/4 h1-FITC pre-mixed with different concentrations of the indicated unlabelled mutants and analysed as in Figure S1D. (F) and (G) hCD40Tg FcγRIIB KO mice that had been adoptively transferred with OTI cells were immunised with 100 μg OVA plus 100 μg of the skewed mutant mAb as in Figure 1. Circulating OTI cells were enumerated over time (mean +/- SD for 5 animals per group) (D) and anti-OVA antibodies in the sera measured on day 18 (E). One of 2 similar experiments shown. *$p<0.05$, ***$p<0.001$.

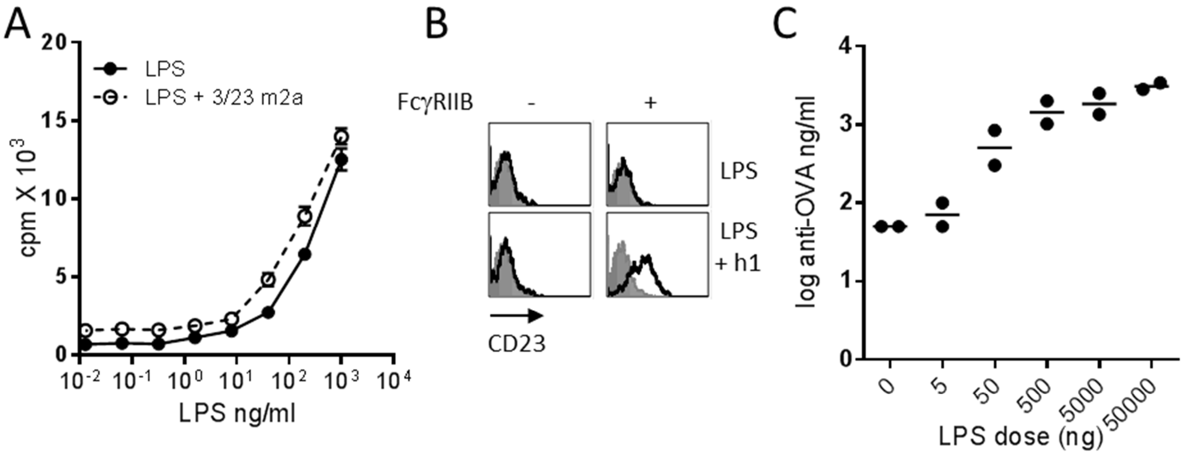

Figure S5. Related to Figure 1. LPS contamination does not account for anti-CD40 mAb activity.

(A) FcγRIIB$^{-/-}$ mouse B cells were incubated with increasing concentrations of LPS in the presence or absence of 400 ng/ml 3/23 m2a. Proliferation was assessed by ^{3}H thymidine incorporation (mean +/- SEM triplicates). (B) Human B-cell activation assessed by CD23 upregulation (black line compared to control, grey) after 16 hr incubation with 1 μg/ml LPS in the presence or absence of 1 μg/ml ChiLob 7/4 h1 and/or FcγRIIB over-expressing cross-linking cells as indicated. (C) Mice were immunised with 100 μg endotoxin-free OVA plus the indicated dose of LPS. Circulating anti-OVA Ab titres were determined on day 14.

SUPPLEMENTAL EXPERIMENTAL PROCEDURES

Chimeric antibody production and quality control

Variable regions were subcloned into expression vectors (pEE6.4 vector for heavy chain and pEE12.4 vector for light chain, Lonza) containing constant regions of different antibody isotypes. Heavy and light chain vectors were further subcloned together before transfection into 293F cells for transient or CHO-K1 cells for stable production of mAbs. Secreted mAb was purified by Protein A-Sepharose (Sigma-Aldrich) chromatography and aggregates (as revealed by SEC-HPLC) removed by gel filtration through Sephadex 200 (Sigma-Aldrich). All preparations were endotoxin low (<1 ng/mg protein) as determined by an Endosafe-PTS portable test system (Charles River Laboratories). Contaminating endotoxin could not account for the mAb functions described in this study as i) mAb concentrations of >10mg/ml would be required to provide enough endotoxin to cause mouse B cell proliferation in vitro (Figure S5A); ii) human B cells do not respond to endotoxin in vitro (Bourke et al., 2003) and Figure S5B), but do show isotype dependent differences in activation with ChiLob 7/4; iii) a dose of at least 50mg of mAb (500-fold that given) would be required to provide enough endotoxin to boost immune responses to this level in vivo (Figure S5C). Flow cytometry and/or SPR were used to assess differences in Ag binding. Non-reducing denaturing capillary electrophoresis (nrCE-SDS) of mAb preparations was performed using a Beckman PA800 Plus analyser according to the manufacturer's instructions. To produce skewed forms of h2, mAb were dialysed into 0.2 M Tris pH8.0 containing 6 mM cysteine plus 1 mM cystamine with (for h2A) or without (for h2B) 2 M guanidine hydrochloride, for 4 days at 4^0C, then dialysed into PBS before use. Pepsin digestion was used to make $(Fab')_2$ fragments that were then chemically reduced to produce Fab' as described (Glennie et al., 1987). Protein A chromatography was used to remove any residual Fc.

Cell isolation, activation and proliferation

Dendritic cells: Human primary Langerhans cells were isolated as described previously (Polak et al., 2012). Briefly, skin specimens were acquired from healthy individuals and epidermal sheets were separated following 20 hr enzymatic digestion (Disopase, 2 IU, Gibco, UK). LCs were harvested following 48 hr migration from epidermal sheets, and enriched to >70% CD1a+ HLADR+ cells by Optiprep™ density gradient (Axis Shield, Norway). Cells were plated into 96 well U-bottom plates at $5x10^4$ cells/well in RPMI 1640 (Gibco, UK) supplemented with Penicillin/Streptomycin (1%, Sigma, UK) and FBS (10%, Invitrogen, UK) and stimulated with ChiLob 7/4 human IgG1 or human IgG2 mAbs or isotype control for 18 h. Expression of activation markers CD40, CD86, CD70 on CD1a+ HLADR+ (all BD Biosciences) LC was assessed by flow cytometry.

B cells: B cells were purified from spleen (mouse) or peripheral blood mononuclear cells (PBMC, human) using magnetic negative selection kits (Miltenyi Biotech or StemCell Technologies). Human PBMC (Lymphoprep, Axis-Shield) were isolated from blood cones obtained from anonymous healthy donors through the National Blood Service (Southampton General Hospital). Cells were plated into 96-well round-bottom dishes at $1x10^5$ cells/well with various concentrations of mAb as described for individual experiments. In some cases, $1x10^5$ 293F cells transfected with human FcγR (White et al., 2011) were also added. To assess activation, cells were photographed (Olympus CKX41 microscope with CC12 soft imaging system) after overnight incubation and activation marker expression analysed by flow cytometry (FACSCalibur, BD Biosciences). Proliferation was assessed by [methyl-^{3}H] thymidine

(PerkinElmer, Cambridge, UK) incorporation after 5 (mouse) or 8 (human) days of culture, as described (White et al., 2011).

T cells: Human PBMCs were labelled with 2 μM CFSE and then pre-cultured for 2 days at high density in 24-well plates as described (Romer et al., 2011), with 1.5 ml of cells at 1 x 10^7 /ml per well. Pre-cultured cells were washed and resuspended at 1 x 10^6/ml for the assay. For some experiments T cells were isolated from pre-cultured PBMCs using a total T-cell isolation kit (Miltenyi Biotec). For the anti-h4-1BB mAbs, wells of 96-well round- bottomed plates were coated with 0.02 μg/ml OKT3 in PBS for 4 h, then washed twice and 10^5 PBMCs /well incubated with 5 μg/ml mAb (final volume 150 μl) for 5 days. Proliferation of $CD4^+$ cells was assessed by flow cytometric analysis of CFSE dilution. For the anti-CD28 mAbs, 10^5 isolated T cells were incubated with mAb in uncoated wells and proliferation assessed as above. Results are expressed as the percentage of divided cells.

For activation of EBV-peptide specific CD8+ human T lymphocytes; HLA-A2 restricted T cells specific for the BMLF-1 epitope of EBV (GLCTLVAML; Cambridge Peptides, UK) were expanded from HLA-A2 individuals as described (Polak et al., 2012). Human primary Langerhans cells (LCs) were incubated with an extended long peptide containing BMLF-1 (proGLC: FNNFTVSFWLRVPKVSASHLEGLCTLVAML,10 μM) for 6h and stimulated with ChiLob 7/4 h1 or h2 mAb or isotype control, 100 ng/ml for 18 h. Pulsed and washed LCs (1x10^4 cells) were co-cultured with BMLF-1-specific T cells (5x10^4 cells) for 20 hours in an ELISpot assay for IFN-γ production (Mabtech, Sweden) as per manufacturer's protocol. Spot forming units (sfu) were enumerated with ELISpot 3.5 reader.

Surface Plasmon Resonance.

A Biacore T100 (GE Healthcare) was used to compare the relative interactions between soluble Fcγ receptors and ChiLob7/4 mAb isotypes. Antibodies or BSA as a reference were immobilised at 15,000 RU to CM5 sensor chips (Biacore) by standard amine coupling according to the manufacturer's instructions. Use of an isotype control mAb to coat the reference flow cell was ruled out due to the presence of Fc. Soluble FcγR (R and D Systems, Abingdon, UK) were injected through the flow cell at 100 nM in HBS-EP+ running buffer (Biacore) at a flow rate of 30 µl/min at 25^{0}C. Regeneration was performed for 30 seconds with 10mM glycine, pH 2. The integrity of the mAb coated onto the flow cells was checked by using positive (hCD40-Fc) and negative (hOX40-Fc) control fusion proteins (R and D systems) at 100 nM (Figure S2C). The background response for the reference flow cell was subtracted automatically; binding to the reference cell was negligible for all FcγR (Figure S2D and data not shown). The integrity of each of the purified FcγR proteins was confirmed by at least one of the following: expected binding profiles for IgG isotypes (White et al., 2011) and this study); increased binding to mAb with mutated Fc known to enhance FcγR interaction (not shown); binding by immobilised anti-FcγR mAb specific for individual FcγR (not shown and Figure S2E). Conditions for the comparison of the interaction between the different anti-CD40 mAb and immobilised hCD40 were as follows: for comparison of Fab' binding, hCD40-Fc (R and D Systems) was immobilised at pH5 at 1000 RU as above and Fab' fragments passed over at 640, 128, 25.6, 5.12 and 1.024 nM (Figure S1E); for comparison of IgG binding, hCD40-Fc was immobilised at 8000 RU and IgG passed over at 100, 20, 4, 0.8 and 0.16 nM (Figure 6F). Regeneration was performed for 30 seconds with 10mM glycine, pH 1.5. Affinities of ChiLob 7/4 h1 and h2 Fab' fragments for CD40 were determined using Biacore Evaluation Software fitting a 1:1 binding model.

SUPPLEMENTAL REFERENCES

Bourke, E., Bosisio, D., Golay, J., Polentarutti, N., and Mantovani, A. (2003). The toll-like receptor repertoire of human B lymphocytes: inducible and selective expression of TLR9 and TLR10 in normal and transformed cells. Blood *102*, 956-963.

Glennie, M. J., McBride, H. M., Worth, A. T., and Stevenson, G. T. (1987). Preparation and performance of bispecific F(ab' gamma)2 antibody containing thioether-linked Fab' gamma fragments. J Immunol *139*, 2367-2375.

Polak, M. E., Newell, L., Taraban, V. Y., Pickard, C., Healy, E., Friedmann, P. S., Al-Shamkhani, A., and Ardern-Jones, M. R. (2012). CD70-CD27 interaction augments CD8+ T-cell activation by human epidermal Langerhans cells. J Invest Dermatol *132*, 1636-1644.

Romer, P. S., Berr, S., Avota, E., Na, S. Y., Battaglia, M., ten Berge, I., Einsele, H., and Hunig, T. (2011). Preculture of PBMCs at high cell density increases sensitivity of T-cell responses, revealing cytokine release by CD28 superagonist TGN1412. Blood *118*, 6772-6782.
